# Space–Air–Ground Integrated Networks for Smart Agriculture and Smart Breeding: A Review of SDN/NFV-Enabled Intelligent Resource Management

**DOI:** 10.3390/s26196181

**Published:** 2026-09-29

**Authors:** Yixiang Zhao, Bo Li, Chenglin Xu, Jiao Zhang, Leilei Wang

**Affiliations:** 1 College of Information and Intelligence, Hunan Agricultural University, Changsha 410128, China; 2 College of Computer Science and Technology, Hengyang Normal University, Hengyang 421002, China; 3 Yuelushan Laboratory, Changsha 410128, China; 4 School of Computer Science and Engineering, Xi’an University of Technology, Xi’an 710048, China; 5 Shaanxi Key Laboratory for Network Computing and Security Technology, Xi’an University of Technology, Xi’an 710048, China

**Keywords:** digital twin, intelligent resource management, network function virtualization, smart agriculture, smart breeding, software-defined networking, space–air–ground integrated network

## Abstract

Smart agriculture and smart breeding are evolving from isolated sensing toward geographically distributed, long-term, data-driven closed-loop management. Dispersed farms and breeding sites, UAV and remote-sensing phenotyping, real-time field control, edge inference, and digital-twin synchronization impose heterogeneous demands on coverage, latency, bandwidth, reliability, computing, and energy. Space–air–ground integrated networks (SAGINs) combine satellites, unmanned aerial vehicles/high-altitude platforms, and terrestrial networks for wide-area coverage and elastic access, but introduce dynamic topologies, heterogeneous multi-domain resources, and complex cross-layer orchestration. Focusing on intelligent SAGIN resource management enabled by software-defined networking (SDN) and network function virtualization (NFV), this review examines controller placement, NFV/service function chain orchestration, SDN/NFV cooperation, learning-driven optimization, and security mechanisms. Representative studies are compared by objectives, decision variables, mechanisms, applicability boundaries, and engineering costs. These mechanisms are connected to smart agriculture and smart breeding through ubiquitous connectivity, edge computing, task offloading, phenotyping, and digital-twin closed loops, while distinguishing experimentally supported agricultural evidence from SAGIN-oriented architectural inference. Future research should move beyond single-metric optimization toward joint evaluation of state freshness, decision latency, reconfiguration cost, learning and security overhead, and agronomic outcomes to improve deployability, robustness, and verifiability of cross-domain resource management under real deployment conditions across heterogeneous agricultural environments.

## 1. Introduction

### 1.1. Definitions and Scope

Smart agriculture and smart breeding increasingly depend on geographically distributed sensing, unmanned aerial vehicle (UAV)-based imaging and phenotyping, field control, edge inference, and digital-twin synchronization. These workloads impose heterogeneous requirements on coverage, latency, bandwidth, reliability, computing capacity, and energy consumption. Space–air–ground integrated networks (SAGINs), which integrate satellite, aerial, and terrestrial infrastructure, provide a useful basis for combining wide-area coverage, mobile access, and hierarchical computing in agricultural environments [1,2,3]. This review therefore uses smart agriculture and smart breeding as its principal application context rather than treating agriculture as one of several interchangeable SAGIN scenarios.

**Figure 1:**
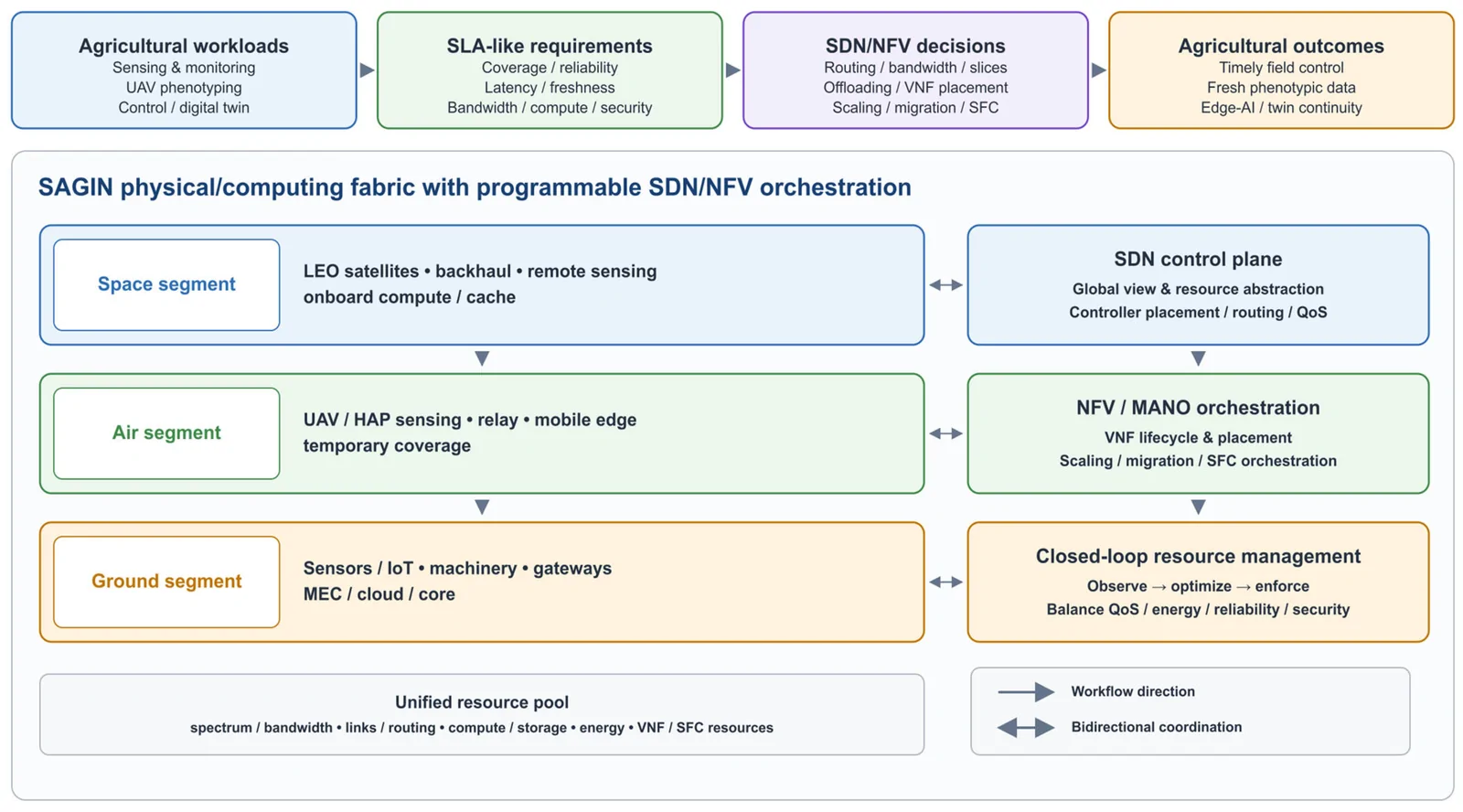
Review-level SDN/NFV-enabled SAGIN framework linking agricultural workloads, service requirements, orchestration decisions, and agricultural outcomes.

For clarity, the principal networking terms used throughout the review are defined here. Software-defined networking (SDN) separates the control and data planes and enables logically centralized, programmable network control [4]. Network function virtualization (NFV) decouples network functions from dedicated hardware and realizes them as virtual network functions (VNFs) on general-purpose computing platforms [5]. Management and orchestration (MANO) frameworks coordinate VNF lifecycle management and resource allocation [6,7], while service function chain (SFC) refers to an ordered sequence of network functions used to deliver a service. In this review, SAGIN resource management denotes the coordinated allocation and reconfiguration of communication, computing, storage, control, and virtualized-service resources across the space, air, and ground segments under quality-of-service (QoS), reliability, energy, and application constraints. Figure 1 summarizes this scope by linking representative agricultural workloads and SLA-like requirements to the SAGIN physical/computing fabric, SDN control decisions, NFV/MANO orchestration, and agricultural outcomes.

Relevant standardization provides the implementation context for these abstractions. The European Telecommunications Standards Institute (ETSI) GS NFV 002 and GS NFV-MAN 001 define the NFV reference architecture and management/orchestration basis [5,6], while the Open Networking Foundation (ONF) OpenFlow protocol provides a standardized programmable-control interface [4,7].

### 1.2. Research Gap, Research Questions, and Contributions

Agricultural sensors, UAV-based imaging and phenotyping, agricultural machinery and irrigation control, edge inference, and long-term digital-twin updates differ markedly in data volume, timeliness, coverage, reliability, and energy constraints. Remote agricultural areas may also face sparse terrestrial infrastructure and limited backhaul capacity. UAV/high-altitude-platform (HAP) access can provide flexible coverage and relaying [8], while satellite coverage and terrestrial edge computing can extend the communication-computing chain across geographically distributed farms and breeding sites [1,2,3]. Existing SAGIN research also demonstrates the broader cross-scenario applicability of this multilayer architecture [2,3,9,10,11]. In this review, however, agriculture remains the longitudinal application thread. Section 9 maps the SDN control, NFV service orchestration, learning-based optimization, and security mechanisms reviewed earlier to ubiquitous connectivity, task offloading, phenotypic sensing, and digital-twin workloads.

The review is guided by four research questions. RQ1: What resource-management pressures arise from SAGIN heterogeneity, topology dynamics, and service diversity, and how do SDN and NFV address them? RQ2: How do controller placement, VNF/SFC orchestration, cooperative SDN/NFV management, learning-driven optimization, and security-aware mechanisms differ in objectives, decision variables, solution methods, reported gains, and engineering costs? RQ3: How can these mechanisms be mapped to agricultural workloads such as heterogeneous connectivity, task offloading, phenotypic sensing, and digital-twin synchronization while preserving the boundary between demonstrated agricultural evidence and architectural inference? RQ4: What unresolved trade-offs and deployment gaps must be addressed for reproducible and deployable SDN/NFV-enabled SAGIN in smart agriculture and smart breeding?

To answer these questions, the main contributions of this review are fourfold. First, driven by the heterogeneous connectivity, edge processing, phenotypic-data flows, and digital-twin synchronization requirements of smart agriculture and smart breeding, we use SDN control, NFV/SFC orchestration, and their cooperation as the principal technical thread to construct a layered review framework spanning control-plane deployment, service orchestration, learning-driven optimization, and security-aware resource management. Second, representative studies are compared in a traceable manner in terms of optimization objectives, decision variables, solution mechanisms, application scenarios, performance gains, and engineering costs, while explicitly distinguishing generic networking evidence, SAGIN-specific evidence, and agricultural-application evidence. Third, smart agriculture and smart breeding are used as longitudinal application scenarios to map ubiquitous connectivity, edge computing, task offloading, phenotypic sensing, and digital-twin requirements onto the SAGIN resource-management chain and thereby assess the deployability of different technical mechanisms against agricultural workloads. Fourth, we synthesize future research directions in onboard intelligence, integrated communication-sensing-computing, digital-twin autonomy, green resource management, distributed management for large-scale low-Earth-orbit (LEO) constellations, and long-term security, emphasizing joint evaluation of network performance, agricultural task timeliness, resource overhead, and deployability.

### 1.3. Organization of the Review

The remainder of this review is organized as follows. Section 3 analyzes the core challenges of SAGIN resource management and motivates the introduction of SDN and NFV. Section 4 focuses on SDN controller placement, including optimization models, clustering-based methods, and intelligent algorithms. Section 5 examines NFV-enabled service orchestration and resource allocation. Section 6 discusses cooperative SDN/NFV resource management. Section 7 and Section 8 review learning-driven optimization and security mechanisms, respectively. Section 9 then uses smart agriculture and smart breeding as representative longitudinal applications to evaluate resource-management requirements for heterogeneous connectivity, edge orchestration, and digital twins in realistic service chains. Section 10 summarizes future research directions and challenges, and Section 11 concludes the review.

## 2. Related Work and Review Positioning

Literature collection followed an iterative, topic-oriented process. We searched and retrieved literature using Google Scholar, Web of Science, IEEE Xplore, ScienceDirect, and arXiv. Searches were conducted in stages around SAGIN, SDN, NFV, and agricultural applications, using combinations of terms including “SAGIN”/“space–air–ground integrated network”, “SDN”/“software-defined networking”, “NFV”/“network function virtualization”, and “smart agriculture”/“smart farming”. The combinations were adjusted across platforms and collection stages; no single fixed Boolean expression was used. No fixed publication-year filter was applied, with relevance to the review scope used as the primary selection criterion. Journal and conference papers were intentionally eligible, while standards/technical reports and arXiv preprints were included when needed to establish technical background or capture emerging developments. The retained corpus spans publications from 2008 to 2026. Topic-focused searches were supplemented by manual screening of reference lists, followed where appropriate by title- or DOI-based retrieval of additional relevant studies.

Broader SDN surveys review control/data-plane decoupling, programmable interfaces, centralized control, and hybrid SDN deployment [12,13]. They provide mechanism-level baselines, but do not address the mobility, heterogeneity, and cross-domain orchestration constraints specific to SAGIN.

Recent SAGIN surveys emphasize complementary dimensions. Xiao et al. review 6G SAGIN architectures and enabling technologies across protocol layers [2]; Chen et al. focus on disaster-management communication and computing requirements [9]; and Hu et al. survey generative-AI-enabled SAGIN security [14]. None is organized around the SDN/NFV resource-management chain examined here.

Table 1 therefore compares the closest research directions by technical focus, representative evidence, unresolved limitation, and relevance to agriculture and SDN/NFV orchestration. The comparison shows a persistent split: networking studies develop programmable control and orchestration without sustained agricultural mapping, whereas agriculture-side reviews emphasize connectivity, edge processing, phenotyping, and digital twins without systematic cross-domain SDN/NFV management.

The unresolved gap is not the absence of SAGIN, SDN, NFV, or smart-agriculture surveys individually. Rather, the surveys compared in Table 1 do not jointly organize controller placement, VNF/SFC lifecycle management, SDN/NFV cooperation, learning and security decisions, and their consequences for agricultural workloads. This review addresses that gap through common comparison dimensions and an explicit distinction among generic networking, SAGIN-specific, and agricultural-application evidence.

Agriculture-side reviews are synthesized in Section 9.1, Section 9.2 and Section 9.3 because they serve a different role from the positioning literature in Section 2. This structure separates literature positioning from application synthesis while preserving the boundary between demonstrated agricultural systems and SAGIN-oriented architectural inference.

## 3. Fundamentals and Challenges of SAGIN Resource Management

**Figure 2:**
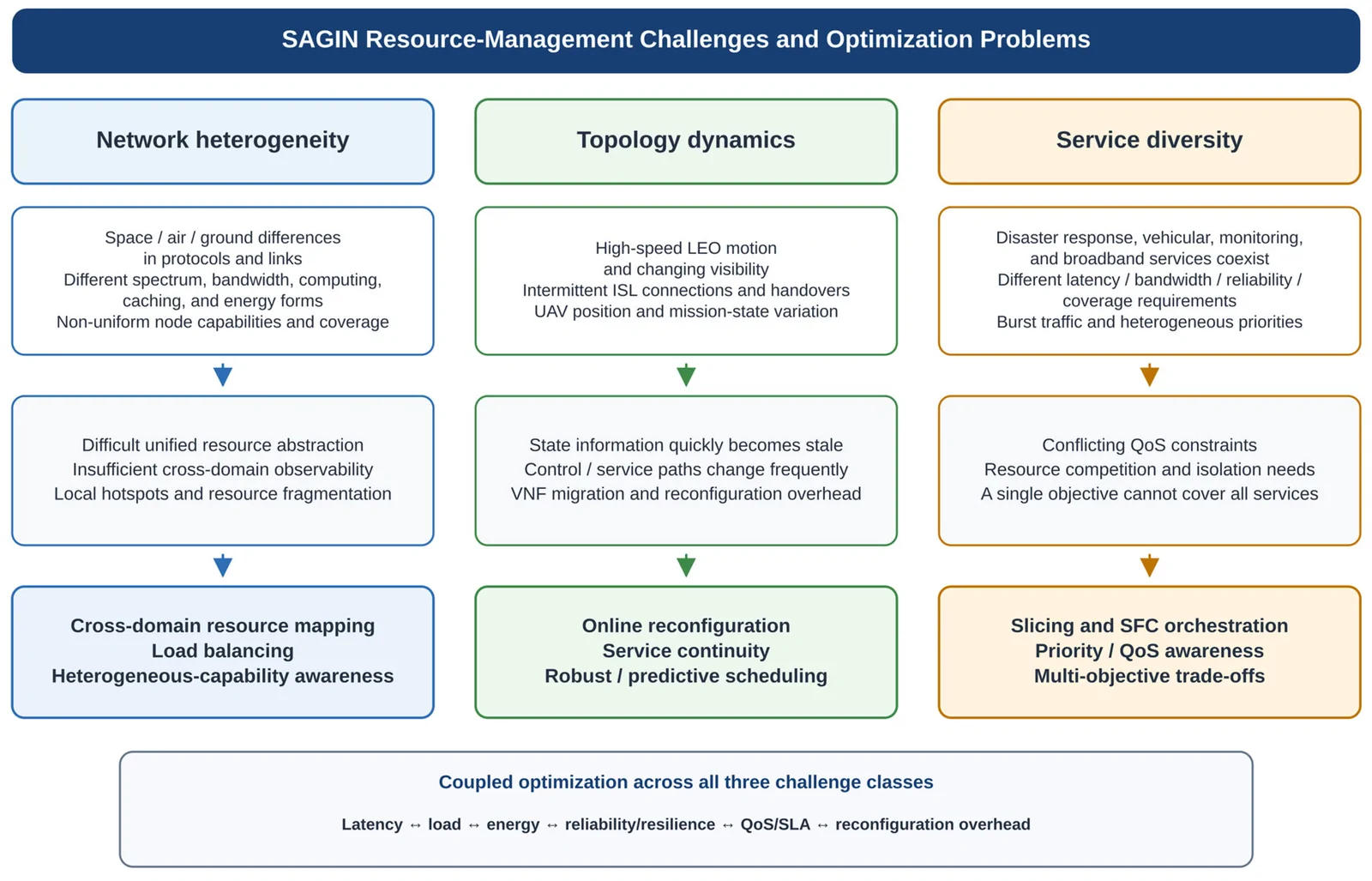
Core SAGIN resource-management challenges and the resulting optimization problems.

After defining the scope and positioning of this review, this section examines the core challenges of SAGIN resource management. We analyze the sources of resource-management pressure in three dimensions: network heterogeneity, topology dynamics, and service diversity. We then explain how these pressures translate into requirements for unified resource abstraction, dynamic scheduling, service continuity, and differentiated QoS assurance. Figure 2 maps these three challenge categories to the corresponding resource-management problems. It also distinguishes them functionally from the overall resource-management architecture in Figure 1.

From a resource-management perspective, the three SAGIN layers are not simply a stack of homogeneous nodes. The space segment provides wide-area coverage and relatively predictable orbital motion, but is constrained by long-distance links, limited onboard computing/storage, and visibility windows. The aerial segment offers rapid deployment and controllable positioning, but is constrained by propulsion energy and wireless backhaul conditions [52]. UAVs also face payload-capacity constraints [53]. The terrestrial segment generally provides stronger computing, storage, and access capabilities, but coverage continuity and infrastructure availability can be scenario-dependent, particularly under disasters. Cross-layer resource management must therefore answer four fundamental questions: where resources are located, when they are available, who controls them, and how they can be reconfigured within service deadlines. Communication, computing, storage, and control capabilities cannot be treated as one static and homogeneous resource pool.

Figure 2 summarizes how heterogeneity, topology dynamics, and service diversity translate into cross-domain resource-management pressures. Table 2 complements this conceptual mapping by decomposing the same pressures at the space, aerial, and terrestrial layers, thereby linking the challenge taxonomy to layer-specific constraints and decisions.

To establish a common language for subsequent sections, we summarize SAGIN resource-management problems using four layers: resources, constraints, objectives, and decisions. The resource layer includes spectrum/bandwidth, power, computing, storage, caching, VNF instances, and controller processing capacity. The constraint layer includes link/node availability, capacity, service-chain order, latency and reliability thresholds, and topology/visibility relationships. The objective layer may target latency, energy consumption, cost, request acceptance, revenue, fairness, security, or learning performance. The decision layer includes node-controller association, routing and bandwidth allocation, VNF placement/migration, task offloading, learning aggregation, and security policies. The key distinction among subsequent sections is therefore not whether they optimize resources, but where their principal decision variables and constraints lie: Section 4 focuses on control-plane placement and association; Section 5 on VNF/SFC orchestration; Section 6 on joint control/orchestration decisions; Section 7 on how learning mechanisms alter the decision process; and Section 8 on incorporating security variables and resource costs into the same evaluation boundary.

Representative studies show why programmable SAGIN resource management must retain lower-layer constraints. Cognitive Ka-band satellite systems must protect incumbent services and manage interference [54], while CubeSat/Internet-of-Space-Things architectures link SDN/NFV orchestration to resource-constrained onboard platforms [55].

Mobility further couples UAV trajectories, visible-LEO selection, battery budgets, uncertain traffic, and satellite visibility. Online multi-armed-bandit approaches reduce dependence on offline training, but handover latency, feature-estimation error, and interoperability remain deployment limits [56].

Cross-domain extension creates additional coordination rather than simply adding links: space–air–ground–sea integration and satellite-enabled 5G both require consistency across access, backhaul, core-network functions, control, and resource abstraction [57,58,59].

Aerial access similarly couples association, power control, interference, mobility, and air-to-ground channel conditions [60,61]. UAV flexibility therefore does not remove terrestrial-access constraints from resource scheduling.

The space segment also couples communications and allocation through beamforming/inter-beam interference, cognitive coexistence, and multiple-access design [62,63,64]. These physical constraints must remain visible to cross-layer control rather than being hidden by an assumption of homogeneous resources.

SAGIN resource management is dominated by three coupled challenges: heterogeneous resources, dynamic topology, and service diversity. Space, air, and ground nodes differ in protocol, computing, energy, and link characteristics; LEO motion and intermittent inter-satellite links continually change associations and backhaul paths [65]; and services span delay-tolerant sensing, latency-sensitive control, and burst data transfer under limited onboard, aerial, and spectrum resources. SDN can provide a unified logical view and programmable policy, while NFV enables service-chain instantiation and migration as topology and workload change [1,2,3,15,66]. These same mechanism classes are mapped to agricultural workloads in Section 9.

SDN/NFV therefore provides the architectural basis for the controller-placement and service-orchestration problems reviewed next. 3GPP non-terrestrial-network specifications and the ITU-R IMT-2030 framework supply an emerging standards context for integrated terrestrial/non-terrestrial deployment [67,68]. Section 4 now examines controller placement and cooperation under dynamic SAGIN topology.

## 4. SDN Controller Placement and Optimization

Different studies select network and computing simulation environments according to the specific tasks of controller placement, service orchestration, and learning-based optimization. The platforms, topologies, traffic models, and parameter settings discussed below therefore follow the experimental configurations reported in the original studies.

### 4.1. Control-Plane Performance Metrics

In SDN-enabled SAGIN, controller placement determines not only the communication distance between controlled nodes and controllers, but also control-request queuing and processing, controller-resource utilization, and the continuity of control paths under failures. Evaluating placement solely through node-to-controller propagation delay cannot fully characterize the control-plane state of a dynamic heterogeneous SAGIN. Based on existing controller-placement studies, this review summarizes control-plane performance along three interrelated dimensions: control-response latency, controller load and processing capacity, and control reliability and resilience. Different studies simplify these quantities according to their target network environments; therefore, the following metrics constitute a unified analytical framework rather than an assumption that every latency or reliability component is equally important in every SAGIN scenario. Broader surveys of the controller placement problem (CPP) likewise show that latency, reliability, load, controller count, and multi-controller coordination should be considered jointly. The three-dimensional framework adopted here can thus be viewed as a SAGIN-focused synthesis for dynamic heterogeneous settings [69].

#### 4.1.1. Control-Response Latency

Control-response latency is a fundamental indicator of the real-time performance of controller placement. Classical CPP formulations primarily consider propagation delay between nodes and controllers. For example, Liao et al. [16] define the propagation delay between node i and controller j at time t as shown in Equation (1):

(1) $$\tau_{ij}^{\mathrm{prop}}(t)=\frac{d_{ij}(t)}{c}$$

where *d_ij_*(*t*) is the physical distance between the node and controller as the topology evolves, and c is the signal propagation speed ([16], Equation (4)). This model is particularly representative for long inter-satellite or satellite-to-ground links. The hierarchical geostationary-Earth-orbit (GEO)/LEO study in [70] further notes that propagation delay is a reasonable scenario-specific simplification when controller-processing capacity is sufficient, queuing overhead is negligible, and long-distance propagation dominates the other latency components. Classical CPP work [71] formulated the average and worst-case node-to-controller delays as k-median and k-center problems, respectively, establishing basic baselines for controller-latency optimization ([71], Equations (1) and (2)).

However, propagation delay does not represent the complete control-response time in SAGIN scenarios with high request-arrival rates, wireless-access contention, or limited controller-processing capacity. Control requests may also experience channel-access delay, link transmission/propagation, controller queuing, and controller processing/service. Abdel-Rahman et al. [72] provide a more complete control-response model for wireless SDN. Using i and j as the node and controller indices for consistency with this review, the M/M/1 queuing and service delay at controller j under aggregated request load can be written as Equation (2):

(2) $$E[D_{q,j}]=\frac{1}{\mu_{j}-\sum_{i}r_{i}x_{ij}}$$

where *r_i_* is the control-request arrival rate of node *i*, *x_ij_* denotes the node-controller association, and *μ_j_* is the service rate of controller *j*. Stable operation requires the aggregate arrival rate to remain below controller-processing capacity ([72], Equation (4)). Abdel-Rahman et al. [73] adopt a similar M/M/1 model for cellular SDN with stochastic request loads, further showing that controller capacity and random traffic arrivals jointly determine control-request queuing/service delay ([73], Equation (3)).

After harmonizing the original notation in [72] with that used in this section, the wireless-controller response time can be expressed as Equation (3):

(3) $$T_{r,ij}=D_{\mathrm{ac}}+2{\tilde{n}}_{ij}t_{ij}+\frac{1}{\mu_{j}-\sum_{i}r_{i}x_{ij}}$$

where *D_ac_* denotes the wireless channel-access waiting time, ${\tilde{n}}_{ij}$ represents the average number of link transmissions after accounting for retransmissions, and *t_ij_* is the one-way communication latency of the node-controller link ([72], Equation (27)). This model shows that control performance over wireless or resource-constrained control links is jointly determined by link access, link communication, and controller queuing/processing.

From a review perspective, SAGIN control latency is therefore better interpreted as a scenario-dependent metric system composed of different physical or logical stages, including transmission, propagation, queuing, and processing. Existing primary studies have not decomposed all four components into four independent analytical expressions within one unified model. For example, *t_ij_* in [72] still combines transmission and propagation effects. This review therefore discusses the relative importance of these components only where supported by existing models, rather than introducing an unsupported four-term analytical decomposition. Propagation delay is typically significant on long satellite links, whereas queuing, processing, and access overhead may become dominant under dense control requests, wireless contention, or limited controller resources. The hierarchical SAGIN controller-placement study in [17] also considers node-controller communication, inter-controller communication, and controller processing in a layered latency evaluation, further demonstrating that propagation delay alone is insufficient for all placement scenarios ([17], Equations (5)–(10)).

#### 4.1.2. Controller Load and Processing Capacity

Controller load reflects the intensity of control requests handled by a controller and is another key determinant of control-plane scalability and queuing performance. Defining load simply as the sum of request rates from managed nodes implicitly assumes that all controllers have identical processing capacity and that all requests consume equal resources. Such assumptions do not always hold in heterogeneous SAGINs composed of terrestrial data centers, aerial platforms, and onboard computing nodes.

From the perspective of request traffic, the aggregate control-request rate received by controller *j* can be written as Equation (4):

(4) $$\lambda_{j}=\sum_{i}r_{i}x_{ij}$$

and should satisfy the basic stability condition in Equation (5):

*λ_j_* < *μ_j_* (5)

where *μ_j_* is the service capacity of controller *j*. As indicated by (2), when *λ_j_* approaches *μ_j_*, controller queuing/service time increases nonlinearly. Capacity constraints therefore do more than prevent nominal overload; they directly affect control-response latency [72,73].

To capture heterogeneous controller resources in SAGIN, ref. [16] constructs a composite load metric that includes control-request pressure, controller capacity, memory status, and central processing unit (CPU) status. To avoid confusion with the reliability threshold beta used later, this review denotes the three weights as *ω*_1_, *ω*_2_, and *ω*_3_, yielding the equivalent form in Equation (6):

(6) $$B_{j}(t)=\omega_{1}\frac{\sum_{i}q_{i}(t)e_{ij}}{Q_{c_{j}}}+\omega_{2}B_{m,j}(t)+\omega_{3}B_{c,j}(t)$$

The first term captures normalized control-request pressure relative to controller-processing capacity, while the second and third terms represent memory and CPU states, respectively; *ω*_1_, *ω*_2_, and *ω*_3_ are the corresponding weights ([16], Equation (6)). Compared with a simple sum of request rates, this formulation captures differences in controller-computing capability and the combined contribution of multiple internal resource states to controller load.

Chiti et al. [74] normalize load according to the buffering and processing capacities of different controller types in an integrated terrestrial/non-terrestrial network and jointly incorporate load and average control latency into the placement objective ([74], Equations (1)–(4)). Zhu et al. [75] use the mean Packet-IN load and its standard deviation to characterize controller load and balancing, jointly evaluating them with disconnection rate and control latency in dynamic SAGIN controller placement ([75], Equations (12)–(14)). Both formulations show that controller-load evaluation in SAGIN should at least distinguish input request pressure, available controller-processing capacity, and load distribution across multiple controllers.

#### 4.1.3. Control Reliability and Control-Path Resilience

Control reliability describes the ability of a node to continue receiving valid control service under component failures, link interruptions, or topology changes. It therefore cannot be equated with the reliability of a single link or path. In SAGIN, high mobility, intermittent connectivity, and layer-specific failure characteristics make control service jointly dependent on node and controller availability, control-path connectivity, multipath redundancy, control-message delay after failures, and backup-path switching capability.

Ros and Ruiz [76] provide a relatively rigorous definition of control reliability from the perspective of southbound control connectivity. For node *i* and its associated controller set *I_i_*, the reliability constraint can be written as Equation (7):

*R*(*G*, *i*, *I_i_*) ≥ *β* (7)

where *R*(*G*, *i*, *I_i_*) is the probability that node *i* can reach its controller set through at least one valid control path after accounting for the operating probabilities of network nodes, links, and controllers, and beta is the minimum reliability requirement ([76], Section 3.1).

Because exact computation of this reliability is costly, ref. [76] further derives a multipath lower bound using disjoint control paths, with the basic form in Equation (8):

(8) $${\hat{R}}_{i}=1-\prod_{p\in P_{i}}(1-\prod_{e\in p}p_{e})$$

where *P_i_* is the set of disjoint control paths from node *i* to its controller set used for reliability estimation, and *p_e_* is the operating probability of the corresponding component in the transformed graph ([76], Section 3.2). Compared with formulations that derive control reliability from link reliability alone, this model jointly incorporates nodes, links, controllers, and multipath reachability and is therefore more suitable as a core definition of control-plane reliability.

Probabilistic reachability alone is still insufficient to characterize post-failure control quality. Guo et al. [77] incorporate the network states induced by node and link failures and their occurrence probabilities into controller placement and optimize control latency under failure states, showing that even when a control path remains connected, rerouting-induced latency degradation can reduce actual control performance. Beheshti and Zhang [78] further study fast failover after failures and use the protection metric in Equation (9):

(9) $$\Gamma \left(T\right)=\sum_{v\in U\left(T\right)}w_{v}$$

Equation (9) characterizes the weighted size of nodes lacking effective protection in a control-routing tree *T*, where *U*(*T*) is the set of nodes without valid fast protection [78]. This metric complements probabilistic reliability models from the perspective of backup paths and rapid recovery.

This review divides SAGIN control reliability into three levels. The first is component reliability, covering nodes, links, and controllers. The second is control-path reliability and multipath redundancy between nodes and controllers. The third is post-failure control continuity, including control-message delay, backup paths, and rapid failover capability. This layered definition avoids equating a reliable link with a reliable control service and more accurately reflects the continuity requirements of dynamic SAGIN control planes.

Latency, load, and reliability are interdependent. Shortening controller-placement distance can reduce propagation delay but may concentrate requests on a small number of controllers. Enlarging controller domains can improve resource utilization but may increase queuing and link-communication delays. Improving reliability usually requires additional controllers, redundant control paths, or reserved backup resources, thereby increasing deployment and management overhead. Controller placement must therefore move beyond single-metric evaluation toward multi-objective or constrained optimization with explicit objective functions and feasibility constraints.

### 4.2. Controller-Placement Optimization Models

Based on the preceding metrics, the controller placement problem (CPP) in SDN-enabled SAGIN seeks to jointly determine the number and locations of controllers and the node-controller association under a given topology, candidate controller locations, control demands, and resource constraints. Because different studies assume different dominant bottlenecks in the SAGIN control plane, no single optimization objective covers all scenarios. Existing models can be grouped according to their main objectives into latency minimization, joint load-latency optimization, capacity-constrained placement, reliability-constrained placement, and failure-resilience optimization.

Let *S* denote the set of network nodes and *C* the set of candidate controllers, and define the binary association variable in Equation (10):

(10) $$x_{ij}=\{\begin{array}{c} 1,\;\mathrm{if}\;\mathrm{node}\;i\;\mathrm{is}\;\mathrm{managed}\;\mathrm{by}\;\mathrm{controller}\;j,\\ 0,\;\mathrm{otherwise}. \end{array}$$

and the controller-deployment variable in Equation (11):

(11) $$y_{j}=\{\begin{array}{c} 1,\;\mathrm{if}\;a\;\mathrm{controller}\;\mathrm{is}\;\mathrm{deployed}\;\mathrm{at}\;\mathrm{candidate}\;\mathrm{location}\;j,\\ 0,\;\mathrm{otherwise}. \end{array}$$

These variables are used only as unified notation for comparing the original models below; each cited study retains its own symbols and definitions.

#### 4.2.1. Latency-Minimization Models

Propagation-delay minimization is the classical form of the CPP. Heller et al. [71] define the average control-latency placement problem as Equation (12):

(12) $$\underset{C^{\prime }}{\min}\frac{1}{\left|S\right|}\sum_{i\in S}\underset{j\in C^{\prime }}{\min}\tau_{ij}$$

and the worst-case control-latency problem as Equation (13):

(13) $$\underset{C^{\prime }}{\min}\;\underset{i\in S}{\max}\;\underset{j\in C^{\prime }}{\min}\;\tau_{ij}$$

which correspond to k-median and k-center controller-placement problems, respectively ([71], Equations (1) and (2)). Here, *τ_ij_* denotes the control-path latency between node *i* and controller *j* as defined in the corresponding study. The formulation is simple and reveals the fundamental relationship between controller location and control-path length, but it does not explicitly model controller capacity, request queuing, failure reliability, or dynamic topology.

Compared with this general CPP baseline, early SAGIN-specific work explicitly introduced SDN controllers into a space–air–ground architecture and investigated dynamic UAV-controller deployment using the maximum and average switch-controller propagation delays as core metrics. Such work provides a scenario-specific starting point for later formulations that further incorporate capacity, load, reliability, and global topology dynamics [79].

For dynamic LEO satellite networks, ref. [18] further incorporates node-controller association into the total communication-latency objective, with the core form in Equation (14):

(14) $$\min\sum_{i\in S}\sum_{j\in C}x_{ij}\tau_{ij}^{\mathrm{comm}}$$

subject to constraints on single-controller association, valid deployment locations, controller capacity, network connectivity, and maximum allowable latency ([18], Equations (17)–(27)). Compared with a pure k-median formulation, this approach more directly captures how dynamic satellite clustering and controller capacity affect placement feasibility.

#### 4.2.2. Joint Latency-Load Optimization Models

When control requests are unevenly distributed, or controller resources differ, minimizing communication distance alone can turn centrally located controllers into performance bottlenecks. Several studies therefore optimize control latency and load balancing jointly. Liao et al. [16] construct separate objectives for propagation delay and controller-load balancing in 6G SAGIN and combine them in Equation (15):

F(*X*) = [*f*_delay_(*X*), *f*_load_(*X*)] (15)

Equation (15) defines a bi-objective controller-placement problem ([16], Equations (8) and (9)). The model shows that placing controllers near many switches can reduce propagation delay while increasing local CPU, memory, and request load, creating an explicit trade-off between the two objectives.

The hierarchical multi-controller model in [17] likewise considers both network-wide average control latency and controller-load imbalance and reduces control overhead through network partitioning and master/slave controller deployment ([17], Equations (11) and (12)). Chiti et al. [74] further address heterogeneous controller-processing resources in integrated terrestrial/non-terrestrial networks by jointly incorporating average control latency and load normalized by controller capacity into the placement cost ([74], Equations (1) and (9)). Hence, the placement that minimizes latency is generally not the same as the placement that maximizes load balance. In highly dynamic SAGIN, more realistic models should explicitly trade off control-path length, request distribution, and heterogeneous control capacity rather than assigning one common capacity constant to all controllers.

#### 4.2.3. Capacity- and Response-Time-Constrained Models

Another class of CPP does not treat latency as the sole objective. Instead, it requires the control-response time to satisfy a service-quality threshold while minimizing the number of controllers or deployment cost. Abdel-Rahman et al. [72] propose a probabilistic response-time-constrained model for stochastic wireless control links, represented by Equation (16):

(16) $$\min\sum_{j\in C}y_{j}$$

subject to the chance constraint in Equation (17):

Pr{*T_r_*_,*ij*_ ≤ *δ*} ≥ *β* (17)

where *δ* is the maximum acceptable control-response time and *β* is the target probability of satisfying this latency requirement ([72], Equations (28)–(31)). Because *T_r_*_,*ij*_ already includes wireless access, link communication, and controller queuing/service effects, this formulation connects controller count, request load, service capacity, and control latency within one optimization framework.

Abdel-Rahman et al. [73] further address stochastic request arrivals using two-stage stochastic programming, whose first-stage objective can be summarized as Equation (18):

(18) $$\min[\sum_{j\in C}y_{j}+E_{\tilde{r}}[h(x,\tilde{r})]]$$

where the first term represents controller-deployment count and the second captures the expected response cost of adaptive node-controller assignment under stochastic request states ([73], Equation (34)). Compared with static CPP formulations based on fixed traffic loads, this class of models is better suited to dynamic networks in which control-request volume varies over time and across user locations.

#### 4.2.4. Reliability-Constrained and Resilience-Oriented Models

In SAGIN, controller placement must also ensure that control service does not completely fail when links, nodes, or controllers are unavailable. Ros and Ruiz [76] therefore include control reliability directly as a CPP feasibility constraint and, while minimizing controller-deployment and association cost, require the constraint in Equation (19):

*R*(*G*, *i*, *I_i_*) ≥ *β*,∀*i* (19)

Equation (19) thereby ensures that each controlled node retains a valid connection to one or more controllers with probability at least *β* [76]. Compared with assigning a reliability threshold to a single link, this formulation explicitly models nodes, links, controllers, and multiple control paths and thus provides a more complete reliability-constrained controller-placement model.

Guo et al. [77] do not merely determine whether the network remains connected; they incorporate different node/link failure states, their occurrence probabilities, and the resulting node-controller control latency into the objective to reduce performance degradation under failures ([77], Equations (5)–(12)). Beheshti and Zhang [78] jointly consider controller location and rapid recovery of the control-routing tree by minimizing the weighted number of unprotected nodes *Γ*(*T*). Both formulations show that reliable controller placement should move from low latency under normal operation toward acceptable control performance under failure states.

#### 4.2.5. Synthesis

Existing CPP models show a clear evolution from single propagation-delay minimization toward joint optimization of latency, load, capacity, reliability, and resilience. Early k-median and k-center formulations reveal the basic relationship between controller location and propagation distance. Later SAGIN-specific work introduces dynamic topology, hierarchical controllers, heterogeneous processing capability, and load balancing. Wireless and cellular SDN studies further show that stochastic request loads and finite controller-service capacity make queuing delay non-negligible, while reliability-oriented work introduces component failures, multipath control connectivity, and failure-recovery capability into the placement model.

This broader objective space also substantially increases CPP complexity. Reducing controller count can lower deployment cost but enlarge control domains and increase communication and queuing delay. Adding controllers and redundant control paths can improve real-time control and fault tolerance but consume additional onboard computing resources and increase synchronization and state-maintenance overhead. Likewise, load balancing may require some nodes to abandon their nearest controller, and reliability enhancement may lengthen control paths. No controller-placement strategy is therefore universally optimal across SAGIN scenarios; optimization objectives should be selected according to network layer, topology dynamics, control-traffic intensity, onboard processing capacity, and reliability requirements.

These models ultimately require concrete solution algorithms. Existing approaches differ substantially in required input information, computational complexity, online adaptability, and global-search capability. The next subsection therefore classifies controller-placement algorithms using a common framework of input, processing, output, application conditions, advantages, and limitations.

### 4.3. Classification of Controller-Placement Algorithms

This subsection classifies algorithms according to their solution mechanisms rather than application scenarios. Given time-varying SAGIN topology, heterogeneous controller resources, and multiple simultaneous objectives and constraints, representative studies are best grouped into four classes: heuristic iterative methods, clustering and network-partitioning methods, metaheuristic and hybrid global-search methods, and exact/stochastic mathematical optimization. Deep reinforcement learning and swarm-intelligence techniques have been explored in the broader SDN controller-placement literature, but direct SAGIN evidence remains insufficient to treat them as major placement paradigms alongside the preceding classes. The taxonomy below is therefore evidence-driven and does not promote under-supported learning-based approaches to the same categorical level.

#### 4.3.1. Heuristic Iterative Methods

Heuristic iterative methods exploit problem structure to construct an initial placement and then improve feasible solutions through local replacement or rule-driven iteration. Bukhari and Song [19] propose Satellite Controller Placement (SCP), using LEO topology, inter-node propagation delay, controller capacity, satellite availability, and historical success rate as inputs. The algorithm first generates an initial controller set according to the average propagation delay of candidate satellites, then exchanges controllers and switches and evaluates each new scheme with a Z-score-type objective, finally outputting the controller count, controller locations, and Open vSwitch (OVS)-controller associations. The method is evaluated over 24 h on an Iridium-like 66-satellite network and includes failure cases in which some satellites cannot host controllers. Its strengths are a transparent procedure and explicit incorporation of capacity and reliability scores, making it a low-overhead approximate baseline for more complex models. Its limitations are that reliability remains an availability-plus-historical-success score rather than a complete control-path failure model and that local exchange does not guarantee a globally optimal solution.

#### 4.3.2. Clustering and Network-Partitioning Methods

Clustering and network-partitioning methods first use node location, distance, traffic, or load features to reduce the candidate search space and then determine controllers within each cluster or subdomain. Bukhari and Song [18] apply K-Means++ to probabilistically initialize cluster centers in a LEO satellite network and combine it with periodic K-Means reassignment. Inputs include orbital parameters, position, connectivity, and traffic load; outputs include control clusters, representative controllers, and time-varying node-controller mappings. K-Means++ reduces instability caused by random initialization and enables re-clustering at discrete intervals as satellites and traffic change. Its main computational cost grows with the number of nodes n, controllers k, and iterations, making it suitable for LEO scenarios requiring periodic updates but unable to afford exhaustive combinatorial search. However, the controller count must still be specified in advance, and repeated clustering incurs overhead. Chen et al. [17] further use an improved K-Means method for network partitioning and controller-count determination in dynamic SAGIN, reducing the size of the subsequent global placement subproblem.

#### 4.3.3. Metaheuristic Search Methods

Metaheuristic and hybrid global-search methods use stochastic acceptance, population search, or memory mechanisms to escape local optima and are well suited to multi-objective, non-convex, combinatorial CPPs. Liao et al. [16] use satellite positions, Packet-IN request volume, controller capacity, and CPU/memory load within each time slot as inputs; combine propagation delay and load variance into a joint loss; and apply simulated annealing to search the controller-switch assignment space. Switch migration is then triggered by dynamic load. The approach can jointly handle latency and heterogeneous resource load and uses the annealing acceptance rule to broaden the search, but repeated candidate evaluation and migration introduce additional runtime and management overhead. For integrated terrestrial/non-terrestrial networks, ref. [74] combines improved simulated annealing with Tabu Search and uses a satellite-visibility threshold to reduce controller switching, producing time-slot-level controller sets, node associations, and a semi-static LEO controller-switching plan. This design is more adaptive to dynamic terrestrial/non-terrestrial topology and heterogeneous buffering capacity, but the CPP remains NP-hard and solution quality depends on iteration budget and parameter settings.

#### 4.3.4. Hybrid Clustering-Global Optimization Methods

Hybrid methods further exploit a reduce-then-search strategy. Chen et al. [17] apply a genetic algorithm after improved K-Means partitioning to search controller locations within each subnetwork, jointly targeting average network latency and load balancing. Inputs include node coordinates, maximum tolerable latency, time-slot distance, and load state; outputs include subnetwork partitioning, the number and locations of master/slave controllers, and switch associations. The method captures hierarchical control, processing latency, and topology dynamics, but the original paper reports complexity O((P + 1)·k·I·n) and notes that GA-based global search takes longer than conventional K-Means/Louvain methods. Zhu et al. [75] use DPC-K-Means for adaptive subdomain partitioning and an improved Snow Ablation Optimizer (ISAO) for master/slave controller assignment. Inputs additionally include local density, relative distance, controller load, and time-slot state; outputs are the subdomains and the globally optimized controller configuration. Chaotic initialization and Levy flights improve the global/local search balance, making the approach suitable for large heterogeneous constellations, but clustering and optimization must be repeated across multiple time slots, so computational cost and parameter sensitivity remain important considerations.

Beyond these approximate approaches, mathematical programming provides auditable exact or stochastic optimization baselines. Abdel-Rahman et al. [72] use candidate controllers, node request rates, controller service rates, wireless-link success probabilities, and response-time thresholds to formulate ILP and chance-constrained stochastic programming models that output controller count, locations, and node associations while constraining response time probabilistically. Abdel-Rahman et al. [73] further use two-stage stochastic programming under random eNB request rates, allowing second-stage associations to adapt to traffic changes. Their strengths are explicit objectives and constraints and the ability to verify optimality or service-quality guarantees, making them suitable for small- to medium-scale offline planning and as benchmarks for heuristics. Their limitations are rapidly increasing computational complexity as the number of variables and scenarios grows. However, refs. [72,73] address wireless/cellular SDN rather than SAGIN-specific networks; they are therefore used here only as general controller-placement baselines, without directly extrapolating their scenario-specific conclusions to satellite networks.

To improve readability, Algorithm 1 presents a survey-level abstraction of the common workflow. It does not rename or reproduce any single paper algorithm; rather, it extracts the shared sequence of state acquisition, candidate reduction, placement search, constraint checking, and dynamic updating from [16,17,18,19,74,75]. The implementation details remain those of the original studies.
**Algorithm 1.** Survey-level common workflow for SAGIN controller placement**Input:** current topology/visibility, service requests and load, candidate controller locations, capacity, and latency/reliability constraints 1: Acquire or update network state and compute inter-node latency, load, and availability features. 2: Reduce the controller-location/association search space using clustering, partitioning, or candidate filtering when applicable. 3: Generate an initial controller set and node-controller association scheme. 4: Update candidate solutions using local iteration, metaheuristic/hybrid search, or mathematical programming. 5: Check capacity, maximum control latency, connectivity/reliability, and controller-count constraints; if infeasible, return to Step 4. 6: Evaluate latency, load, fairness, reliability/resilience, deployment cost, and other objectives or metrics. 7: If the stopping condition is satisfied, output controller locations and associations; otherwise continue the search. 8: In dynamic scenarios, repeat state updating and local/global adjustment when topology, traffic, or failure-state thresholds are triggered. **Output:** controller number/locations, node associations, and required migration/switching plans.

As summarized in Table 3, no controller-placement algorithm dominates across all SAGIN scenarios. Heuristic methods emphasize lower implementation complexity and interpretability. Clustering and partitioning improve scalability by compressing the search space. Simulated annealing, genetic algorithms, ISAO, and hybrid search can address more complex multi-objective placement problems but require more iterative computation. Mathematical programming provides clear optimality and constraint baselines but is more sensitive to problem scale. More importantly, online applicability in dynamic SAGIN depends not only on theoretical algorithmic complexity but also on topology-update intervals, controller-migration frequency, state-collection overhead, and onboard computing capability. Future algorithm design should therefore emphasize the joint trade-off among solution quality, runtime, and control overhead rather than judging methods by latency alone. Learning-based resource-management approaches such as deep reinforcement learning (DRL) and multi-agent reinforcement learning (MARL) are currently more common in broader resource allocation, slicing, and service-orchestration problems and are discussed separately in Section 7.

As discussed later in Section 9, controller placement may have application-level deployment implications for geographically distributed farms and breeding sites, where UAV sensing and real-time field control depend on timely state collection. Topology updates, control latency, controller migration or switching, and reliability/resilience could therefore affect agricultural task timeliness and the risk of acting on stale control state. The primary controller-placement studies reviewed in Section 4 do not constitute direct agricultural experimental evidence; they provide a basis for application-level mapping to these requirements. Controller placement determines control-plane management locations and associations, whereas on-demand service instantiation and migration still require fine-grained NFV orchestration; Section 5 turns to NFV-enabled SFC orchestration and resource optimization.

## 5. NFV-Enabled Service Orchestration and Resource Optimization

By decoupling network functions from dedicated hardware and deploying them as VNFs, NFV provides a foundation for unified abstraction, elastic scaling, and on-demand migration of heterogeneous SAGIN resources. Service function chaining (SFC) further organizes multiple VNFs into ordered processing chains according to service logic. Resource management therefore becomes more than a single-node selection problem; it couples VNF placement, link routing, execution order, resource capacity, service latency, and migration/recovery decisions. Based on the existing literature, Section 5 is organized into three levels: basic models and problem formulation, classification of solution mechanisms, and comparison of representative methods and section boundaries. The traceable analytical framework used in Section 4 for objectives, constraints, algorithmic mechanisms, application scenarios, and limitations is retained.

### 5.1. NFV/SFC Fundamentals and Problem Formulation

NFV/SFC resource management does not have a single universal minimum-cost objective. Existing SAGIN studies typically trade off four categories of quantities: (1) end-to-end service performance, including deployment latency, completion time, and QoS constraints [20,21,22,23,80,81,82,83,84]; (2) resource consumption and cost, including computing, bandwidth, storage, and VNF instantiation [20,21,22,23,80,81,82,83,84]; (3) migration, re-instantiation, and failure-recovery overhead in dynamic environments [21,23,82]; and (4) system utility, such as request acceptance, long-term revenue, or service-provider profit [20,21,22,23,80,81,82,83,84]. Accordingly, when specific objective functions are discussed below, we also explain the physical meaning of their terms and their relationship to capacity, sequential execution, flow conservation, and service-continuity constraints rather than forcing objectives from different studies into a single weighted formulation.

As an upper-layer resource-organization background, network slicing creates mutually isolated logical networks on shared physical infrastructure to support differentiated services and relies on SDN/NFV for programmable control and virtual-resource abstraction [85]. More recent surveys on slicing and cloud-native networking extend this view to end-to-end resource management, VNF/CNF placement, and MEC cooperation [86,87,88]. In this review, network slicing is therefore treated as a service- and resource-organization context for SFC/VNF orchestration rather than as generic 5G evidence that substitutes for SAGIN-specific primary studies.

In ultra-dense LEO access networks, slicing decisions are directly affected by the spatiotemporal variation of users and visible satellites. Existing work combines eMBB/uRLLC resource slicing, throughput, resource utilization, and reconfiguration cost into a long-term utility and uses DRL for dynamic slicing decisions, demonstrating direct coupling between satellite-slicing resource management and learning-based optimization [89].

The architectural relationship can be stated more precisely. SDN is well suited to resource abstraction and programmable control, but slice lifecycle management, resource management, and orchestration still require NFV/MANO capabilities. SDN, NFV, and slicing should therefore be understood as a cooperative relationship among control abstraction, virtual resources/lifecycle management, and service-level logical networks, rather than as three parallel concepts [90].

Cross-timescale coupling has become an explicit issue in satellite-edge service orchestration. One line of work combines small-timescale constrained Markov decision process (CMDP)/Lyapunov/soft actor–critic (SAC) scheduling with large-timescale service deployment in a hierarchical iterative framework [91]. Another uses long-term service deployment to constrain the feasible action space of short-term scheduling and jointly optimizes dynamic service deployment and task scheduling through a dual-timescale framework [92]. Both approaches show that optimizing service placement and real-time scheduling separately can miss cross-timescale feedback, although the resulting gains still depend on the assumed time windows, traffic statistics, and topology prediction.

This cross-timescale structure also raises the question of when to re-optimize part of the orchestration problem. If long-term service placement changes too frequently, image/state migration, path reconstruction, and control-signaling overhead are amplified. If placement is updated only at fixed long intervals, the system may continue using stale deployments after substantial changes in topology, traffic, or resource load. References [91,92] demonstrate through hierarchical iteration and dual-timescale constraints, respectively, that long-term deployment and short-term scheduling cannot be completely separated. At the survey level, it is therefore more useful to distinguish planning-level deployment, service-level scheduling, and event-triggered recovery and to examine whether explicit information feedback exists among these timescales rather than comparing only the objective value within a single time slot.

For large-scale satellite-edge systems, centralized orchestration also faces the cost of global-state collection and signaling. Distributed service-deployment work uses ADMM to jointly optimize computing, communication, and storage resources and treats dependence on centralized global information as a major limitation [93]. Future SAGIN orchestration should therefore evaluate not only where a service is placed but also the scope and freshness of the state required for the decision and the cost of distributed coordination.

For mission-critical services, SAGIN-specific edge-intelligence work has placed service-demand decomposition, microservices/lightweight virtualization, cross-domain cooperative control, and joint communication–computing–caching allocation within an end-to-end service lifecycle [94]. Engineering service-management experiments for 5G/B5G, meanwhile, provide deployment-oriented references for scaling, dedicated slicing, and failure-triggered migration [95]. The two lines of work show that service continuity requires joint treatment of heterogeneous resources, migration/failures, and cross-domain control-state synchronization overhead.

From a lifecycle perspective, VNF/SFC orchestration includes distinct operations such as instantiation, scaling, placement/re-placement, migration, failure recovery, and termination, each with different requirements for state information and execution deadlines. Initial deployment depends primarily on service arrivals, resource capacity, and path reachability; scaling requires continued observation of load variation; migration must jointly consider source and destination resources, state transfer, and service interruption; and failure recovery must connect detection, candidate redeployment, and service reconnection into a continuous process [21,23,82,94,95]. A successful VNF placement therefore establishes only a feasible mapping at one instant and does not demonstrate complete lifecycle management. For dynamic SAGIN, more meaningful evaluation should also record the frequency of instance adjustments, the number of migration/recovery operations, state-synchronization volume, service-interruption windows, and cumulative cost of repeated reconfiguration.

From the broader perspective of network softwarization, slice resource management also involves energy efficiency, MEC resource slicing, and O-RAN-based aerial–terrestrial integration. Surveys have already treated energy efficiency as an important slice-lifecycle metric; MEC work further examines edge-resource slicing; and O-RAN aerial–terrestrial networks for maritime services illustrate the extension of slicing into heterogeneous coverage environments [96,97,98]. NFV/SFC orchestration in SAGIN should therefore consider isolation, resource efficiency, and cross-domain implementability rather than comparing only one-shot mapping gains.

SFC mechanisms themselves are also evolving from chain placement toward joint design of in-chain traffic steering and edge execution location. General surveys on SFC traffic steering emphasize path selection and policy enforcement within service chains, while satellite-edge VNF-placement studies directly consider execution locations under satellite-edge resource constraints [99,100]. These studies help distinguish three different but coupled decision layers: logical service order, physical routing, and VNF-instance location.

As a traceable baseline model, Zhang et al. [20] formulated latency-sensitive SFC mapping using deployment-path latency prediction. Total deployment latency consists of VNF-processing delay and link-transmission delay. The latency expression and its minimization objective are given in Equations (20) and (21), respectively:

(20) $$TD_{q}=\sum_{v_{m}\in N}DT_{m}+\sum_{e_{i,j}\in L}DT_{i,j}$$

(21) $$\min\;T\;D_{q}$$

subject to the user-latency constraint *TD_q_* ≤ *UD_q_* and to node and link resource consumption not exceeding their respective capacities ([20], Equations (4)–(8)). This model is useful as an interpretable baseline for this section, but it is not equivalent to later models for profit maximization, resource balancing, recovery-latency minimization, or long-term lifecycle optimization. The discussion below is therefore organized around problem objectives, solution mechanisms, and dynamic adaptability rather than around an artificial unified objective that does not exist in the primary literature.

First, for dynamic VNF mapping and scheduling, Li et al. [21] jointly formulate VNF mapping and execution scheduling after the arrival of new services in an SDN/NFV-enabled vehicular SAGIN. Their mixed-integer linear programming (MILP) model jointly considers VNF mapping, execution order, end-to-end latency, additional latency and migration cost caused by live VNF migration and re-instantiation, and service-provider cumulative profit as the primary objective. Because solving the exact MILP online is costly, the authors develop two Tabu Search algorithms, TS-MAPSCH and TS-PSCH, to obtain near-optimal solutions. TS-MAPSCH permits migration and re-instantiation and therefore has a more flexible search space, but this flexibility also introduces migration cost and additional delay, creating an explicit trade-off between profit and reconfiguration overhead [21].

Second, heterogeneous SAGIN resources couple QoS satisfaction with the need to avoid local resource depletion. Zhang et al. [80] formulate SFC embedding for network slicing as an ILP and propose multidimensional weighted SFC mapping (MDW-SFCM). Rather than selecting only the shortest path, the method evaluates both path-use frequency and real-time residual capacity among feasible paths and uses dynamic weights to reduce overuse of hotspot links and satellite nodes. With 1000 dynamic user requests, the paper reports a 25.17% reduction in user rejection rate and a 6.79% improvement in satellite-resource balance relative to PW-SFCM [80]. These results support treating resource balance as an independent performance dimension of SFC orchestration rather than evaluating deployment solely by average latency.

When service demands are multi-attribute and scenario dependent, Tian et al. [24] avoid simple shortest-path or resource-cost ranking. They construct scenario recognition and a multicolor-set representation, convert resource scheduling among vehicular users, user groups, and SAGIN devices into a three-sided matching problem, and develop SoRSA and VoRSA stable-matching algorithms. Relative to the baselines, the study reports average improvements of approximately 7.4% in effective acceptance rate, 6.3% in cost efficiency, 8.3% in demand–resource matching degree, and 15.3% in overall network performance, together with an approximately 3.7% reduction in end-to-end latency [24]. This class of methods shows that, when multidimensional QoS requirements are explicit, matching stability and demand–resource compatibility can become design objectives that are distinct from conventional cost minimization.

For multicast services, Sun et al. [81] study multicast SFC (MSFC) embedding in heterogeneous SAGIN, jointly formulating VNF placement and multicast routing as an ILP to increase network revenue while reducing computing and bandwidth costs. They also develop polynomial-time heuristics for the NP-hard problem. The study explicitly differentiates the scarcity and cost coefficients of resources across network layers; however, its topology is treated as quasi-static within each analysis window. It is therefore better regarded as representative evidence for heterogeneous-resource-aware multicast embedding than as an online migration solution for rapidly changing topology.

When nodes or related links fail, SFC resource management becomes a problem of service continuity and recovery latency. Jia et al. [82] build an SFC failure-recovery model using a reconfigurable time-expanded graph (RTEG) and decompose the total completion time of an SFC into transmission, processing, storage, VNF redeployment, and additional waiting caused by the redeployment of other SFCs. Their ILP minimizes total processing and redeployment time subject to unique VNF placement, sequential execution, flow conservation, and binary-decision constraints. A matching-game-based FRMG-SAGIN recovery algorithm is then developed. The paper reports an approximately 25% reduction in total time consumption relative to the baseline while also noting that large-scale applicability requires further study and that the method requires a trade-off between latency gain and computational overhead [82].

From the perspective of recovery triggering, dynamic orchestration must distinguish necessary migration from repeated reconfiguration caused by transient state fluctuations. Online migration in [21] adds delay and cost; [82] shows that failure-recovery search itself incurs computational overhead; and [23] explicitly incorporates lifecycle-wide migration into long-term maintenance. Together, these studies support an important deployment boundary: a trigger should not depend only on instantaneous link or load changes, but should also consider remaining service time, failure persistence, expected benefit, and migration cost. Thresholds, hysteresis, and minimum residence times have not been standardized across the existing literature, so they are not presented here as established algorithms. Instead, suppressing migration oscillation and avoiding situations in which short-term gains are offset by repeated migration cost should be treated as mandatory reporting issues for future dynamic-SFC evaluation.

At the higher slice level, resilience in satellite–terrestrial edge networks also requires cross-domain redundancy reservation, SLA decomposition, and coordinated failure detection and recovery. Service recovery should therefore not be understood solely as local redeployment of one SFC but should extend to service-continuity assurance at the multi-domain slice level [101].

Another source of dynamics is contention among SFCs caused by bursty tasks. Jia et al. [22] use an RTEG to represent topology and resource states across time slots, formulate dynamic SFC scheduling as an integer linear programming (ILP) problem that maximizes the number of successfully deployed SFCs, convert the problem to an MDP, and solve it with DRL. This illustrates that learning methods in this section do not simply replace all mathematical programming; rather, they provide approximate online solutions for temporally evolving, competitive, high-dimensional decision problems.

For more complex learning-driven orchestration, Mo et al. [83] propose a cost-aware enhanced time-expanded graph (C-eTEG) that represents transmission, computing, storage, and transceiver resources in a unified temporal graph and jointly formulates VNF placement and routing as an ILP. A D3QN policy maximizes long-term service-provider profit in a dynamic state space, while adversarial reinforcement learning improves robustness to interference and state perturbations. DyFLO [23] combines a time-aggregated graph, GAT-GRU spatiotemporal feature extraction, actor–critic DRL, pseudo-VNF sharing, and lifecycle-wide migration to explicitly address heterogeneous QoS, topology evolution, and long-term maintenance. The paper reports that DyFLO improves system revenue by 35.3% over TMSM and 106.6% over PSO, while increasing effective service capacity by 53.7% and 80.4%, respectively [23].

Federated learning has also been used directly for SFC embedding. Zhang et al. [84] propose FL-VNFE, in which local agents in each domain learn node features and a global agent performs reward-based aggregation, while node load and SFC reconfiguration are considered to reduce blocking. Relative to CLF, the paper reports improvements of 12.9% in long-term average revenue, 2.52% in SFC-request acceptance, and 10.5% in long-term average revenue-to-cost ratio [84]. The authors nevertheless note that the current SFC scheduling mechanism still relies mainly on simple re-embedding and that migration under abnormal failures remains future work. This study therefore provides evidence for learning-based SFC embedding in Section 5, while Section 7 discusses only its federated-learning mechanism, distributed training structure, and privacy/communication overhead to avoid counting the same contribution twice.

### 5.2. Classification of SFC Deployment and Orchestration Algorithms

Based on the preceding studies, SFC solution mechanisms in SAGIN are more appropriately divided into four categories: exact mathematical-programming baselines, heuristic and search methods, matching/game-theoretic methods, and learning-driven dynamic orchestration. This taxonomy is based on the actual solution mechanism and avoids placing all non-DRL approaches into a generic heuristic category.

#### 5.2.1. Exact Mathematical-Programming Baselines

The main value of exact mathematical programming is not that it can directly support every online orchestration problem, but that it explicitly represents constraints on VNF placement, link selection, execution order, capacity, latency, and service continuity and thereby provides an auditable feasible region and a performance upper bound or benchmark for approximate algorithms. References [20,80,81,82] construct ILP/MILP models for latency-sensitive SFC mapping, resource balancing, multicast embedding, and failure recovery, respectively, showing how similar binary placement and routing variables lead to different constraint sets as service objectives change. Their strengths are explicit objective semantics and the ability to identify constraint violations and benchmark heuristics against optimal or near-optimal solutions. Their main limitation is a rapidly increasing variable count as the number of nodes, links, time slots, and SFCs grows. In dynamic SAGIN, mathematical programming is therefore better suited to offline capacity planning, small- to medium-scale windowed optimization, or algorithm benchmarking than to the assumption of full global re-optimization after every topology change.

#### 5.2.2. Heuristic and Search Methods

Heuristic and search methods trade solution quality against online complexity by reducing candidate sets, designing neighborhood moves, or introducing resource-aware weights. The Tabu Search in [21] searches for higher-profit solutions through mapping adjustments, migration, and re-instantiation, but a larger neighborhood also means greater search overhead and migration cost. Zhang et al. [80] use path-use frequency and residual capacity to construct dynamic weights that proactively avoid resource hotspots. Sun et al. [81] develop polynomial-time heuristics for NP-hard multicast-SFC embedding. These methods have practical advantages: they require no long-term training, are relatively interpretable, and allow search depth to be adjusted according to network scale. Their outcomes, however, depend strongly on candidate generation, neighborhood design, weighting, and stopping criteria. Under rapidly changing topology, algorithm runtime must also be evaluated together with the validity period of the state on which the decision is based. In other words, a lower mapping cost has deployment value only when the underlying state remains valid at decision completion and reconfiguration overhead is acceptable.

#### 5.2.3. Matching and Game-Theoretic Methods

When local preferences or utilities can be defined among resource owners, service requests, and candidate nodes, matching and game-theoretic mechanisms can avoid direct enumeration of global combinations. Tian et al. [24] use three-sided stable matching to represent compatibility among vehicular demands, user groups, and SAGIN resources, making demand–resource matching quality and stability evaluation dimensions distinct from shortest path. Jia et al. [82] use a matching game to reduce the search complexity of failure recovery, while the potential-game VNF-placement work in [100] provides adjacent evidence for converging toward stable configurations through local utility iterations. These approaches are suitable for distributed or multi-party resource selection, but stability or equilibrium does not imply global optimality. Performance depends on whether the preference functions realistically capture latency, residual resources, migration cost, and topology lifetime. Survey comparisons should therefore report convergence/stability properties, required information scope, and resulting resource efficiency rather than treating the existence of an equilibrium as sufficient evidence of effectiveness.

#### 5.2.4. Learning-Driven Dynamic Orchestration

Learning-driven approaches are primarily intended for scenarios in which topology, service arrivals, and resource states evolve continuously and conventional combinatorial optimization cannot be repeatedly solved within the decision window. Jia et al. [22] convert dynamic SFC scheduling into an MDP and use DRL to increase the number of successful deployments. Mo et al. [83] apply D3QN to cost-aware temporal VNF placement and routing and use adversarial training to improve robustness to state perturbations. DyFLO [23] combines spatiotemporal feature extraction, actor–critic decision-making, VNF sharing, and lifecycle-wide migration. FL-VNFE [84] uses cross-domain local learning and aggregation to reduce dependence on centralized training. Learning methods can incorporate historical state and long-term return into online policies, but their costs include training samples, model-update communication, inference latency, distribution shift, and cross-topology generalization risk. Section 5 therefore evaluates how these methods solve SFC business problems and their orchestration gains, while Section 7 compares the learning mechanisms themselves, including state/action spaces, training-execution structures, and federated communication overhead, thereby avoiding double counting the same work as two independent contributions. Table 4 summarizes this comparison.

NFV-enabled SFC resource management has expanded from one-shot static mapping to dynamic rescheduling, resource balancing, multicast embedding, failure recovery, and lifecycle optimization. This evolution is not a simple linear replacement from mathematical programming to heuristics to DRL. The methods are complementary: exact models provide constraint baselines, search and matching methods reduce online complexity, and learning methods address temporal dynamics.

The deployability of the methods in Section 5 can therefore be evaluated through a chain that is more complete than mapping quality alone. First, the state requirement describes the scope and freshness of the topology, resource, and service state needed by an algorithm. Second, decision latency asks whether a solver, matching process, search method, or learning inference can produce a decision before that state becomes stale. Third, reconfiguration cost includes VNF instantiation, state migration, path updating, and service interruption. Finally, service continuity asks whether the service remains feasible under latency, capacity, and reliability constraints after execution. Exact optimization, heuristics, matching, and learning differ not only in objective values but also in how complexity and overhead are distributed across these four stages. Only by reporting state requirements, decision time, reconfiguration cost, and service continuity together can the suitability of an SFC orchestration method for highly dynamic, resource-constrained SAGIN be assessed.

Potential agricultural functions such as phenotype preprocessing, disease recognition, image and data fusion, and digital-twin synchronization can be mapped onto NFV/SFC lifecycle properties including placement, migration, state freshness, reconfiguration cost, service interruption, and service continuity. This application mapping may help define workload-aware orchestration objectives, but it does not by itself establish improved phenotyping performance. Complete NFV/SFC deployment for smart breeding remains an architectural mapping rather than established end-to-end experimental evidence, consistent with the evidence boundary in Section 9.4. On this basis, Section 6 focuses on joint decision-making and cross-layer coupling between SDN global control and NFV/VNF mapping, while Section 7 focuses on the learning mechanisms themselves, including DRL, federated learning, and multi-agent learning, without repeating the service problem and algorithmic contribution of the same SFC paper.

## 6. Cooperative SDN/NFV Resource Management

The essence of SDN/NFV cooperation is not the simple coexistence of two technologies, but a closed loop of network-state sensing, virtual-resource abstraction, service instantiation, path scheduling, and feedback updating. SDN controllers aggregate cross-domain topology, link, and resource states to provide a programmable global network view. NFV decouples functions such as firewalls, deep packet inspection (DPI), and network address translation (NAT) from dedicated appliances and implements them as dynamically deployable VNFs, allowing service requirements to be translated further into node selection, VNF instantiation, and path-orchestration decisions. The optimization object thus expands from controller location or single-SFC mapping to joint decisions across cross-layer states, resources, services, and policies. Virtual network embedding (VNE) is a representative cross-layer resource-mapping problem in this context. Existing studies indicate that cooperation can be understood at least through information sharing, resource abstraction, joint mapping, and dynamic closed-loop control. These are not mutually exclusive categories, but implementation levels reflecting increasing SDN/NFV coupling depth. In the broader NFV/VNE literature, virtualized resource abstraction and VNE have long been treated as central problems of mapping nodes, links, and functions onto shared infrastructure [7,102]. Such studies are used here only to define the conceptual boundary of SDN/NFV cooperation; specific SAGIN mechanisms are analyzed from the SAGIN primary studies discussed in this section.

### 6.1. Cooperation Mechanisms and the Boundary of Joint Decision Making

From the perspective of joint objectives, SDN/NFV cooperation involves at least four coupled components: service-side QoS/QoE or request-acceptance benefit; infrastructure-side CPU, bandwidth, and path feasibility; dynamic-service-side VNF migration, re-instantiation, and operational expenditure (OPEX); and control-side signaling and computation introduced by state acquisition and cross-domain information sharing. References [21,24,25,26] combine these dimensions differently, so cooperation cannot be summarized by a single fixed objective function. Each representative model below is therefore explained in the order of optimized benefit, resource/service constraints, and dynamic reconfiguration cost before its solution mechanism is discussed.

Under relatively loose coupling, SDN primarily performs state acquisition and network abstraction, while NFV or virtual-network algorithms use topology, CPU, bandwidth, and latency information supplied by the controller to perform resource mapping. The QoS-aware multi-domain VNE study by Zhang et al. [25] is representative. Infrastructure providers need not expose all internal information to SDN; instead, they upload basic states such as topology, node CPU, intra-/inter-domain link bandwidth, and latency at each time instant to reduce signaling and computational overhead. On this basis, SAGOI-Net is abstracted as a hierarchical physical network containing space, aerial, and terrestrial/maritime domains, while virtual-network requests specify computing, bandwidth, and latency demands. Here, the principal value of SDN is observability and abstraction, whereas VNE remains the responsibility of independent learning and path-selection algorithms.

Tighter coupling directly embeds real-time SDN state into VNF/SFC decisions. The dynamic VNF mapping and scheduling scheme in [21] re-adjusts VNF mapping and execution order when new IoV services arrive and explicitly includes the migration cost and additional latency caused by live migration and re-instantiation. Its MILP maximizes cumulative service-provider profit, while TS-MAPSCH and TS-PSCH provide online approximate solutions. In this case, network state supplied by the controller is no longer merely an offline input; it continuously triggers service remapping and scheduling changes. The three-sided stable-matching scheme in [24] further includes SAGIN devices, user groups, and vehicular users within the same matching process and uses scenario recognition and multi-attribute resource descriptions to construct demand–resource compatibility, thereby exposing multi-party coupling among service requirements, network resources, and service mapping.

In hierarchical closed-loop cooperation, the control plane and VNF execution locations are explicitly assigned to different layers. In LVS-STIVNs [26], an SDN controller on a GEO satellite aggregates global topology and real-time resource states; MEO/LEO satellites perform OpenFlow forwarding; and VNF instances are hosted on OVSs in LEO. The network-management center handles service interaction and overall orchestration, while the control layer determines forwarding paths and the sequence of VNF traversal. This structure moves beyond the conventional division in which satellites only transport traffic and NFV is deployed solely on the ground, allowing SDN control, satellite forwarding, and NFV service instances to form a cross-layer closed loop. The original system architecture also explicitly shows bidirectional relationships among the control layer, data layer, virtualized resource pool, and SFC deployment.

**Figure 3:**
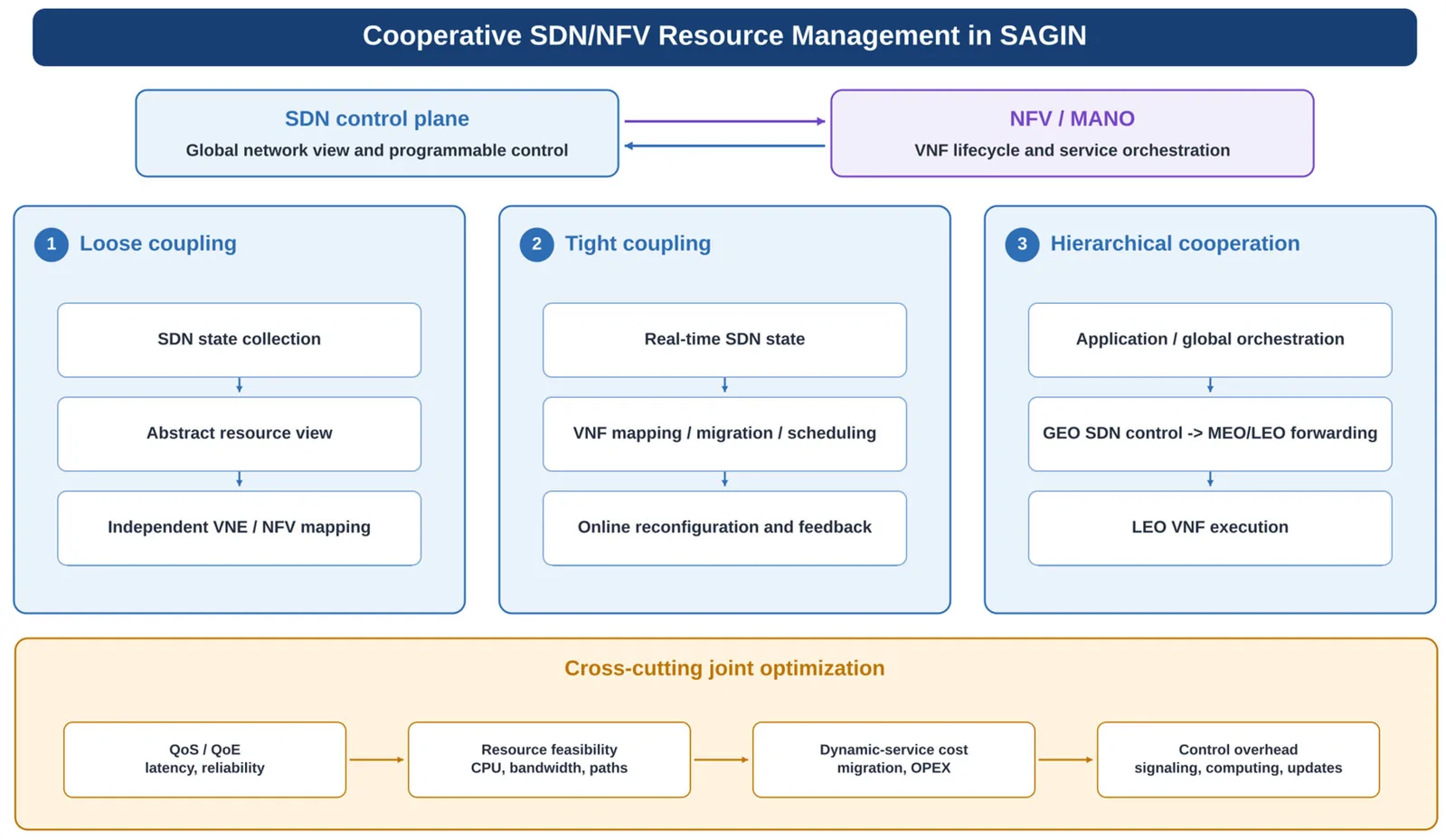
Taxonomy of cooperative SDN/NFV resource management in SAGIN and the cross-cutting joint-optimization property.

To remain consistent with the taxonomy adopted in this review, these mechanisms can be organized through loose coupling, tight coupling, hierarchical cooperation, and joint optimization as four analytical views. This mapping is a survey-level organizational framework rather than a renaming of the original schemes. Loose coupling corresponds to information-level cooperation such as [25], where the control plane supplies cross-domain state, and the virtualization algorithm independently performs mapping. Tight coupling corresponds to cases such as [21], where VNF migration, re-instantiation, execution order, and online network state are embedded in one mapping/scheduling problem. Hierarchical cooperation is clearest in LVS-STIVNs [26], with explicit separation among application management, GEO control, MEO/LEO forwarding, and NFV execution in LEO. Joint optimization is not a separate fourth architectural category; rather, it describes the problem property that multiple cross-layer variables are optimized simultaneously, as in the profit–migration–latency coupling of [21], multi-party demand–resource matching of [24], and joint QoE/QoS/OPEX reward in [26]. The terms coupling architecture and optimization-property are therefore kept distinct throughout this review. Figure 3 illustrates the three coupling architectures and the cross-cutting joint-optimization property.

From a deployability perspective, a cooperative closed loop first faces a joint choice of state granularity and state freshness. Continuously collecting fine-grained states for every node, link, and VNF can improve mapping and reconfiguration decisions, but under fast SAGIN topology dynamics, state collection, aggregation, and dissemination themselves consume link and controller resources, and part of the state may already be stale by the time a decision is completed. Zhang et al. [25] ask infrastructure domains to expose only necessary topology, CPU, bandwidth, and latency information, trading reduced information exposure for a usable global view. Li et al. [26], by contrast, place real-time bandwidth, latency, and VNF state directly into an online MDP, representing higher-frequency and finer-grained control/orchestration coupling. Neither is universally superior; they occupy different points between lower control overhead with coarser state and stronger dynamic response with higher synchronization cost.

Three timescales can be distinguished within this cooperative loop. The underlying resource pool, control hierarchy, and candidate VNF locations typically belong to a relatively slow planning layer. Service arrivals, VNF selection, routing, and bandwidth allocation operate at an intermediate orchestration timescale. Link availability, queue congestion, and satellite-visibility changes may require still faster local response. Li et al. [21] trigger mapping, migration, and re-instantiation on service arrivals, while [26] selects VNF instances sequentially from online state. These studies show that globally re-optimizing every variable at one common frequency is not the only possible design. A more implementable engineering approach is to keep slow variables relatively stable and adjust medium- and fast-timescale variables only when thresholds, service events, or topology changes are triggered, thereby reducing signaling and computation caused by continuous global reconfiguration.

Cooperative design must also specify who generates a decision, who executes it, and who performs rollback after failure. In loose coupling, the SDN controller behaves primarily as a state provider and path executor while VNE/NFV algorithms independently produce mapping results. In tight coupling, controller state directly affects VNF migration and re-instantiation. Hierarchical cooperation separates application management, GEO control, MEO/LEO forwarding, and LEO VNF execution [26]. A single optimization result must therefore be decomposed at implementation time into control rules, instance-lifecycle operations, and forwarding updates. If any step cannot be executed because of link interruption or resource shortage, the resulting state must be fed back to the orchestrator for feasibility re-evaluation. Complete cooperation thus requires not only joint optimization but also decision delivery, execution confirmation, and failure rollback.

### 6.2. QoS-Aware Multi-Domain Virtual Network Embedding

As an adjacent-system baseline, multi-server MEC has already jointly modeled task offloading, uplink power, and server computing resources and exposed its dependence on global task/channel/server states and additional backhaul signaling [103]. SAGIN adds cross-domain visibility, mobile topology, and state-freshness constraints. Having more jointly optimized variables is therefore not equivalent to having a deployable cross-domain closed loop.

Evidence closer to non-terrestrial networks shows that satellite–terrestrial MEC requires joint selection of offloading nodes, offloading ratios, and computing resources, and may share satellite-computing capacity through inter-satellite links [104]. In air–ground MEC, a service instance must first be deployed or initialized before a task has a feasible execution location, so service placement, access/offloading, and resource allocation exhibit ordering constraints and long-term coupling [105]. These results reinforce the central boundary of this section: service orchestration and task scheduling should be viewed as mutually constraining components of one loop rather than independent modules.

The underlying multi-domain VNE problem is not unique to SAGIN. Classical studies have examined how substrate path splitting/migration affects embedding and how coordinated node–link mapping should be performed [106,107]. Meanwhile, surveys of MEC task offloading show that offloading is itself jointly constrained by task granularity, wireless/computing resources, and latency [108]. Comparing these baselines with SAGIN primary work clarifies the distinction between combinatorial difficulties inherent to VNE/offloading and additional difficulties introduced by space–air–ground heterogeneity, mobile topology, and cross-domain state sharing.

Zhang et al. [25] use resource-feasibility constraints and performance metrics for multi-domain VNE rather than a fixed linearly weighted objective. If virtual node n^v is mapped onto physical node n^s, the physical CPU must be no smaller than the virtual-node computing demand. If a virtual link is mapped onto a physical link, available bandwidth must satisfy the requested bandwidth and physical-link latency must not exceed the allowed virtual-link latency. Each virtual node can be mapped to only one physical node. Together, these constraints define the multi-domain VNE feasible region through computing, bandwidth, latency, and unique mapping, as summarized in Equation (22).

(22) $$\begin{array}{cc} \mathrm{CPU}(n^{s}) & \geq \mathrm{CPU}(n^{v})\\ \mathrm{BW}(n_{i}^{s},n_{j}^{s}) & \geq \mathrm{BW}(n_{m}^{v},n_{n}^{v})\\ D(n_{i}^{s},n_{j}^{s}) & \leq D(n_{m}^{v},n_{n}^{v})\\ {\mathrm{Num}}_{\max}(n^{v}) & =1 \end{array}$$

For objective design, ref. [25] does not use one fixed linear combination. Instead, it dynamically selects reinforcement-learning rewards according to the QoS type. For high-bandwidth or high-computing-demand requests, the agent uses the ratio between virtual-network revenue and resource cost as the reward. For low-latency requests, it uses the ratio between aggregate bandwidth and embedding delay. The paper also defines the QoS-dependent reward forms in Equation (23), together with long-term average revenue, long-term revenue-to-cost ratio, and VNR embedding delay to evaluate both resource efficiency and service performance. Thus, the optimization objective in SDN/NFV cooperation need not be one universally weighted function; it can switch according to service QoS class.

(23) $$\begin{array}{cc} r_{R/C} & =\frac{R(G^{v},t)}{\mathrm{Cost}(G^{v},t)}\\ r_{\mathrm{delay}} & =\left[\frac{\sum_{e^{v}\in L^{v}}\mathrm{BW}(e^{v})}{\mathrm{Delay}(G^{v},t)}\right]\beta \end{array}$$

The algorithm follows a cooperative sequence of classification, state extraction, node decision, and link embedding. K-means first divides VNRs into computing-, bandwidth-, and latency-oriented demand classes. Features such as CPU, node degree, adjacent-link bandwidth and latency, and average distance are then extracted from physical nodes to form a state matrix that is continuously updated as resources change. The policy network outputs the probability of mapping each virtual node to each physical node, and k-shortest paths are finally used for intra- and inter-domain virtual-link embedding. A request is rejected if no candidate path simultaneously satisfies bandwidth and latency constraints. The principal benefit is that resource preference can adapt to QoS type rather than using the same fixed objective for all VNRs.

Experimental results further illustrate the effect of the dynamic QoS reward. For low-latency requests, RL-QoS-VNE reduces embedding delay by 17.14%, 17.29%, and 40.81% relative to SA-RL-VNE, RL-VNE, and BaseLine, respectively, with corresponding VNR-acceptance improvements of 2.7%, 2.6%, and 8.5%. Under high-bandwidth/high-computing demand, its revenue-to-cost ratio is higher by 1.1%, 1.4%, and 23.7%, and its acceptance rate by 1.3%, 2.7%, and 8.5% [25]. The original paper also notes that candidate physical nodes are prefiltered to ensure sufficient computing resources, which can leave some resources idle, and identifies more realistic feature extraction and automatic QoS-demand recognition as future work. The method is therefore best viewed as an example of QoS-adaptive VNE supported by SDN state rather than as a universal model for all SDN/NFV joint-optimization problems.

### 6.3. Control/Orchestration Closed Loop for Dynamic SFCs

Li et al. [26] provide tighter control/orchestration coupling than [25]. In LVS-STIVNs, the GEO controller centrally acquires link-state and resource data, MEO/LEO nodes perform only OpenFlow forwarding, and LEO nodes additionally host VNFs that can be instantiated on demand. For an SFC containing N network functions, the request selects one of M_i candidate VNF instances in each service segment t_i. A candidate may be an already running instance or a new instance launched on a node with sufficient resources. The latter introduces additional startup time and OPEX from virtual machine and VNF operation. Whether to instantiate a new VNF and how to select the path therefore become coupled service-orchestration decisions.

The study does not reduce cooperation to one link-cost objective. Instead, SFC deployment is defined as maximizing end-to-end QoE subject to multidimensional QoS constraints. Positive QoS metrics such as bandwidth must meet lower bounds, negative metrics such as latency must not exceed upper bounds, and exactly one VNF instance must be selected for each network function; these constraints are summarized in Equation (24).

(24) $$\begin{array}{cc} \underset{c\in C}{\max}{\mathrm{QoE}}_{c} & \\ \mathrm{subjectto}\;{\mathrm{QoS}}_{c}^{t} & \geq {\mathrm{QoS}}_{\mathrm{con}}^{t},\;t\leq K\\ {\mathrm{QoS}}_{c}^{t} & \leq {\mathrm{QoS}}_{\mathrm{con}}^{t},\;t>K\\ \sum_{j}x_{ij} & =1 \end{array}$$

To make these constraints amenable to online learning, ref. [26] formulates dynamic SFC deployment as an MDP. The state includes available bandwidth, link latency, bandwidth/latency constraints of each VNF, the cumulative latency of the current SFC, and the index of the VNF currently being deployed. The action selects one reachable candidate VNF instance. The resulting Markov decision process (MDP) links observed network state to sequential orchestration decisions. The immediate reward, given in Equation (25), has three components: QoE gain, a QoS-constraint penalty, and an OPEX penalty. Real-time resource state observed by the controller thus enters the policy through the MDP state, NFV-instantiation cost enters through OPEX, and user experience and service constraints enter through QoE gain and QoS penalties, forming a typical cross-layer closed loop.

(25) $$r_{c}={\mathrm{gain}}_{c}^{\mathrm{QoE}}-{\mathrm{pen}}_{c}^{\mathrm{QoS}}-{\mathrm{pen}}_{c}^{\mathrm{OPEX}}$$

At the solution level, rDQN-SFC replaces part of the feature-processing stage at the end of a deep Q-network (DQN) with a long short-term memory (LSTM) module so that each decision can exploit a continuous history rather than a single snapshot. The original experiments report median QoE values 2.02, 2.09, and 2.78 times those of Monte Carlo tree search (MCTS), deep deterministic policy gradient (DDPG), and DQN, respectively, while constraint-violation/error rates decrease by 51.2%, 51.1%, and 72.5%. Relative to exhaustive search, QoE is reduced by only 13.4% while response speed improves by 96.4%. When SFC arrival rate increases from 100 to 240 per time unit, error rate rises by only 2.6%, and QoE falls by 14%. When SFC length increases from 5 to 15, error rate rises by 4%, and QoE falls by 18.6%, while mean response time remains almost unchanged [26]. These results support online dynamic orchestration, but the paper also notes that maintaining longer LSTM histories increases inference latency relative to other DRL models and that fixed QoE weights do not adapt well to different vehicular-service priorities. The study should therefore be interpreted as a closed-loop example combining control state, NFV instantiation, and QoE/QoS rewards rather than simply as a generic DRL algorithm for Section 7.

### 6.4. Comparison of Cooperative Methods and Section Boundaries

Comparing [21,24,25,26] shows that the key difference among SDN/NFV cooperation schemes is not the specific solver but where the two technologies interact in the decision chain. In [25], SDN primarily supplies cross-domain resource awareness, and a learning algorithm performs VNE. Li et al. [21] place dynamic service arrivals, VNF migration/re-instantiation, and scheduling in one MILP and Tabu Search process. Tian et al. [24] organize multidimensional service requirements, device resources, user groups, and vehicular users into a three-sided matching process. Li et al. [26] further close controller state, VNF instantiation, QoS/QoE, and dynamic policy learning within one MDP. From the perspective of cooperation intensity, the evidence can therefore be summarized as state sharing, joint mapping/scheduling, multi-party resource matching, and closed-loop dynamic orchestration rather than forcing all studies into one mathematical objective.

Greater cooperation depth does not guarantee unconditional performance improvement. More frequent state sharing gives the controller a finer-grained resource view, but cross-domain reporting increases signaling and computational overhead. The fact that [25] requires infrastructure providers to upload only essential topology, CPU, bandwidth, and latency information already reflects an observability–overhead trade-off. After online migration and re-instantiation are introduced, ref. [21] explicitly shows that mapping flexibility competes with migration cost and additional latency. Closed-loop learning adds further training and inference costs. Although rDQN-SFC in [26] achieves high QoE and low constraint-violation rates, the authors note that LSTM histories increase inference latency, while the matching scheme in [24] shifts complexity toward demand representation, preference construction, and multi-party stable matching. Evaluation of SDN/NFV cooperation should therefore include state-synchronization frequency, control overhead, VNF-migration cost, decision latency, scalability, and adaptation speed to dynamic topology, not only final revenue, latency, or acceptance rate.

These observations also show that centralized control plus virtualization is not automatically equivalent to true joint optimization. A complete control/orchestration loop exists only when SDN-observed state actually changes NFV-side instance selection, resource mapping, migration, or path decisions and when execution results are fed back into subsequent control cycles. If SDN only provides a static topology snapshot and NFV is solved independently offline, the system is closer to information-sharing loose coupling. If the controller continuously senses resource changes and triggers VNF remapping, rescheduling, or new instantiation, it reaches decision-level tight coupling. This distinction is especially important in SAGIN because satellite-visibility windows, cross-domain link capacity, and service arrivals are all time-varying. Cooperative algorithms must balance more timely global state against lower control/computational overhead. Coupling location, feedback closure, and online cost are therefore the main comparison axes in this section, while the internal learning/solution mechanisms of DRL, FL, and matching are left to Section 7 or their relevant algorithmic sections.

State-synchronization frequency is not a quantity to maximize blindly. Updates that are too slow create stale state and may lead a controller to select VNFs using obsolete link capacities or node loads. Updates that are too fast increase telemetry, control messages, and orchestration computation and may even retrigger reconfiguration before the preceding migration has stabilized. Existing literature does not provide one synchronization period suitable for every SAGIN scenario. A more robust evaluation therefore reports the relative relationship among state-acquisition period, decision/execution time, topology-change rate, and service-completion time rather than only final algorithmic gain. This is particularly important in dynamic constellations: online cooperation is meaningful only when sensing, computation, and rule delivery complete substantially within the validity window of the critical state.

Control/orchestration consistency is another often-overlooked deployment cost. VNF remapping or new instantiation is commonly accompanied by path switching, flow-table updates, and service-state migration. If forwarding rules in the network and VNF-instance state in NFV are not updated consistently, transient unreachability, duplicate forwarding, or service-state inconsistency may occur. Li et al. [21] include migration and re-instantiation cost in online SFC scheduling, while [26] restricts action feasibility through candidate-instance connectivity and QoS constraints. These mechanisms show that a cooperative algorithm must determine not only where the service should move next, but also how path and service-chain continuity are preserved before and after migration. This review therefore treats execution consistency and migration-stabilization time as deployment dimensions alongside control latency and OPEX, even though the existing literature does not report them using one standardized metric.

From a scalability perspective, cooperative schemes exhibit a structural trade-off between centralized observability and distributed autonomy. Centralized control facilitates a unified cross-domain resource view and global service policies, but as the number of satellites, UAVs, users, and VNF instances increases, so do state matrices, candidate mappings, and control messages. Hierarchical or local decision-making can constrain the state size within one domain but must maintain consistency across domain summaries, boundary resources, and local objectives. Zhang et al. [25] reduce internal information exposure through domain-level abstraction, while [26] separates global management from forwarding/VNF execution through hierarchical control. These correspond to two scalability strategies: information compression and responsibility layering. Future comparisons of cooperative architectures should therefore report the amount of state maintained by controllers, the volume of cross-domain messages, and the scope of nodes involved in a single reconfiguration to determine whether performance gain is purchased through non-scalable global information collection.

The evaluation framework of this section can thus be summarized along four interrelated axes. The state axis assesses observable-state granularity, update frequency, and stale-state risk. The decision axis asks whether SDN state actually changes VNF instances, paths, migration, and resource allocation. The execution axis examines whether rule delivery, instance-lifecycle operations, migration/switching, and failure rollback form a closed loop. The cost axis jointly reports signaling, computation, migration, OPEX, and decision latency. Only when all four axes are addressed can merely using SDN and NFV be distinguished from forming deployable cooperative SDN/NFV resource management. The same framework maps directly to smart agriculture: changes in UAV coverage, edge-node load in the field, or the priority of phenotypic/image services constitute cross-layer agricultural cooperation only if they update state and actually trigger service and path reconfiguration.

Agricultural workloads also differ in bandwidth, latency, reliability, computing demand, and freshness: periodic sensing, emergency pest or disease alarms, UAV imagery, phenotype preprocessing, and digital-twin updates do not impose the same service profile. At the architectural level, these requirements suggest a cooperation chain from agricultural service semantics to SDN policy, routing and resource allocation, and NFV service placement or migration. This mapping is a design basis, not a validated agricultural SAGIN implementation. Table 5 summarizes the representative cooperative mechanisms and their trade-offs.

These comparisons also define the boundaries of subsequent sections. Section 6 focuses on how SDN observability and control policies are coupled with NFV/VNF mapping at the information and decision levels. Even when [25,26] use reinforcement learning, their role here is to show how learning connects control state with virtualized orchestration. Section 7 discusses only learning-mechanism issues such as state–space design, training structure, distributed coordination, and generalization for DRL, MARL, and FL, avoiding repetition of the same SFC/VNE service model. Security, privacy, and VNF-isolation risks remain in Section 8. This separation creates a progression from the control plane in Section 4, to NFV/SFC orchestration in Section 5, to joint cooperation in Section 6, and to intelligent decision mechanisms in Section 7 rather than overlapping treatments.

## 7. Learning-Driven Intelligent Resource Optimization

The methodological spectrum of intelligent resource management should also include supervised deep learning [109]. Multi-cell radio-resource-allocation studies have used training labels generated by optimization algorithms to learn mappings for joint subband and power allocation, showing that online inference can replace part of the high-complexity iterative optimization when training data sufficiently cover the operating distribution [110]. However, effectiveness depends on the training distribution, label quality, and scenario generalization, so such methods should not be directly compared with online reinforcement learning using a single performance metric.

At the methodological foundation, DQN stabilizes deep Q-learning through experience replay and target networks and therefore provides an original anchor for value-based DRL in network-resource control [111]. QMIX, in turn, combines multi-agent value functions through a monotonic mixing network under centralized training with decentralized execution (CTDE), while its original formulation explicitly notes representation limits under partially observable Dec-POMDP settings [112]. These foundational limitations help avoid attributing MARL performance gains simply to a larger number of agents.

In direct air–ground–space cooperative computing, SkyLink jointly optimizes AirX deployment location, collaborative offloading, and communication/computing resources using mixed discrete-continuous multi-agent actions [113]. Such work indicates that the value of learning methods lies mainly in near-real-time approximation of dynamic hybrid decisions, while scalability remains constrained by agent count, state sharing, and changes in the training distribution.

Beyond SAGIN-specific learning algorithms, edge-intelligence research has developed a multilayer ecosystem ranging from edge deep learning and end-edge/cloud collaborative computing to digital twins, federated learning, and swarm-learning cooperation [114,115,116]. These surveys show that model placement, data/model movement, and the hierarchy of computing resources are themselves system-design variables. Intelligent resource management therefore cannot be evaluated solely by the reward achieved by a policy network.

The privacy properties of federated learning also require more precise wording. Blockchain-FL research in industrial IoT attempts to enhance traceability and privacy in distributed data sharing; differentially private deep learning provides formal privacy mechanisms; and FL surveys systematically summarize communication, statistical heterogeneity, and optimization challenges [117,118,119]. Keeping raw data local should therefore be interpreted as reducing centralized raw-data collection rather than automatically eliminating leakage from model updates, system heterogeneity, or training overhead.

For reinforcement learning, work ranging from dynamic uplink/downlink resource allocation in high-mobility 5G heterogeneous networks and experience-driven network control to MARL benchmarks for C-V2X resource allocation continues to expose the influence of state–action–space size, multi-agent non-stationarity, partial observability, and policy robustness/generalization [120,121,122]. These adjacent studies reinforce the comparison boundary adopted here: the value of DRL/MARL lies in online decision capability, but applicability still depends on state observability, training samples, agent scale, and the communication cost of coordination.

**Figure 4:**
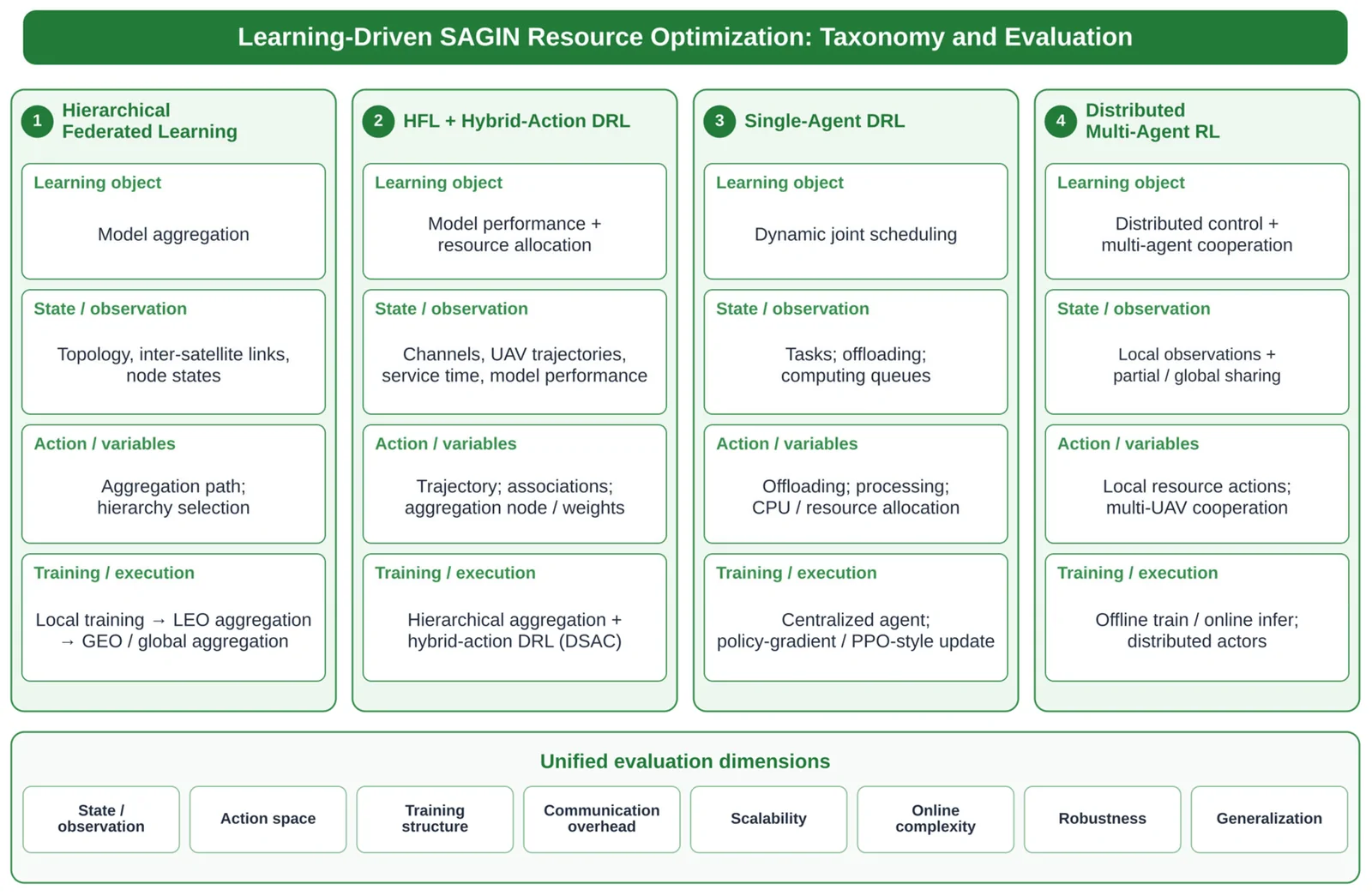
Taxonomy and unified evaluation dimensions of learning-driven SAGIN resource optimization.

Unlike the preceding sections, this section does not focus on one service-mapping problem or control architecture, but on how learning mechanisms change the decision process of SAGIN resource management. Representative work can be divided into at least four distinct technical routes: hierarchical/federated learning for model aggregation, hierarchical federated learning (HFL) combined with DRL for hybrid control variables, policy-gradient DRL for single-agent continuous decisions, and MARL for distributed coordination. This distinction matters because using AI does not imply identical state spaces, training procedures, or system overhead. Applicability is instead determined by the learning object, the scope of information sharing, the structure of the action space, and the training-execution mode. To provide a common background, this review also draws on surveys of federated learning (FL) aggregation and heterogeneity [123], FL for 6G edge intelligence [124], communication constraints in wireless FL [125], DRL methods for communications and networking [126], DRL for slicing and virtualization [127], digital-twin networks and simulation evolution [128,129], and MARL for B5G slicing resource allocation [130]. These sources define general boundaries among learning paradigms, whereas performance claims for SAGIN algorithms in this section are based on experiments reported in SAGIN primary studies. Figure 4 classifies the methods according to learning object and decision structure and lists common evaluation dimensions.

The objectives of learning mechanisms should likewise be decomposed. Rewards or losses in existing SAGIN work typically combine task/model performance, communication or computing cost, energy consumption, task-drop or reliability penalties, and fairness constraints [27,28,29,30,31]. FL/HFL emphasizes trade-offs among model accuracy, aggregation energy, and communication reliability. Single-agent DRL commonly incorporates latency, energy, and task completion into system cost or reward, while MARL additionally introduces multi-executor cooperation and penalties for constraint violations. The discussion below therefore first clarifies what each algorithm actually optimizes before introducing states and actions, and then asks whether higher reward is accompanied by greater training, communication, or information-sharing cost.

From the general constraints of wireless edge systems, distributed learning extends beyond FL to federated distillation, distributed inference, and MARL. Their deployment is jointly constrained by dynamic channels, spectrum/power limits, and heterogeneous terminal computing capabilities [131]. These general constraints are used to define the communication-computing boundary of SAGIN learning mechanisms; concrete performance claims remain grounded in SAGIN primary studies.

### 7.1. Hierarchical and Federated Learning: From Data Locality to Model Aggregation Across Space, Air, and Ground

The basic federated-learning paradigm trains models locally without centrally uploading raw data. Participants submit model updates to a server, and a shared model is formed through aggregation. Frequent model updates therefore introduce communication rounds and bandwidth overhead as a core engineering constraint [132].

Collaborative federated learning can further reduce dependence on a single central controller and incorporate communication mechanisms such as network formation, device scheduling, mobility management, and coding into learning-performance optimization, showing that the FL architecture itself can become a wireless resource-management design variable [133].

The Hierarchical Learning and Computing framework proposed by Zhu et al. [27] is fundamentally a multilayer federated/hierarchical-learning system rather than a reinforcement-learning resource-allocation method. Terrestrial IoT devices train local models using local data, LEO satellites collect and aggregate local models over inter-satellite links, and GEO satellites handle routing management and global-model broadcast. The global learning objective remains standard weighted empirical risk minimization, as shown in Equation (26):

(26) $$\underset{x\in R^{d}}{\min}f(x):=\sum_{n=1}^{J}\lambda_{n}f_{n}(x)$$

where *λ_n_* is the aggregation weight of device n and *f_n_*(*x*) is its local loss. The new resource-management problem arises in model-aggregation communication rather than in the learner itself. The authors divide predictable satellite topology into snapshots, use model-transmission energy on each directed inter-satellite link as edge weight, and formulate aggregation routing in each frame as the minimum-energy directed Steiner tree problem in Equation (27):

(27) $$P_{2}:\;\min W_{u}(B(m,u))=\sum_{i,j\in V}w_{i,j}(m,u)$$

Because the Directed Steiner Tree (DST) problem is NP-hard, the paper proposes TAEER. Dijkstra shortest paths are first computed from each terminal LEO satellite to the root, after which a Chu–Liu–Edmonds procedure is applied on an alternative directed graph to find a minimum spanning arborescence. Complexity is dominated by repeated Dijkstra searches and is *O*(|*V′*|(|*V*| + |*ε*|*log*|*ε*|) + |*ε_s_*|*log*|*V_s_*|) [27]. Thus, the central issue in this form of learning-resource optimization is not designing a state–action–reward tuple, but converting model traffic produced by FL into a network-optimization problem constrained by topology, energy, and reliability.

The experiments demonstrate why this distinction matters. In a Walker–Delta constellation, TAEER reduces energy consumption by as much as 3.64% and 67.50% relative to D-Merge and Orbit-Greedy, respectively, while reducing average link-interruption probability by 30.82% relative to D-Merge. Under the 800/20/1 configuration, solving P2 requires approximately 0.027 s, far below the 10 s frame duration [27]. The trade-off is dependence on available topology snapshots and inter-satellite-link state. The authors further extend the analysis to unpredictable events such as link interruptions, satellite failures, and congestion and explicitly study the energy-reliability trade-off. The key bottleneck of hierarchical FL in SAGIN is therefore often not local SGD itself but the model-aggregation path, ISL reliability, and onboard energy consumption.

A deeper integration route is the HFL resource-allocation framework of Huang et al. [28]. UAVs serve as edge aggregators, LEO satellites as cloud aggregators, and satellites can continue exchanging models through ISLs. Unlike fixed FedAvg, the system jointly optimizes UAV trajectories, ground-user-to-UAV association, UAV-to-LEO association, the final aggregation satellite, and model weights at the edge, cloud, and final aggregation stages. The objective is not simply to maximize the accuracy of one task, but to improve average performance across multiple FL tasks within limited satellite-service time while maintaining inter-task fairness.

Because the problem contains mixed discrete and continuous variables, the authors use Distributional Soft Actor–Critic (DSAC) and handle the hybrid action space through a decouple-and-recouple strategy. The state *s*(*t*) contains ground-to-air channel coefficients, UAV trajectories, remaining service time of space–air links, and current test performance at UAV/LEO aggregators. The action *a*(*t*) jointly includes trajectories, multi-level access associations, final aggregation-node selection, and aggregation weights [28]. This differs fundamentally from generic DRL-based resource allocation because the learner must simultaneously control wireless connectivity, mobility, and model-aggregation rules, and separately learned continuous and discrete actions must be recombined through a hybrid policy.

In the experiments, H-DSAC achieves an average test accuracy of 91.4% at 100 s of communication time, compared with 90.4%, 85.6%, 84.9%, and 81.9% for four baselines. When all ground users transmit at 0.2 W, H-DSAC reaches approximately 97.99%, versus 96.4%, 94.1%, and 94.2% for three baselines. With eight non-independent and identically distributed (non-IID) tasks, H-DSAC still reaches 96.9%, while the baselines achieve approximately 91.7–95.2% [28]. The hybrid action space, however, makes training substantially more complex than standard continuous- or discrete-action DRL. The authors note that post-deployment prediction overhead is far smaller than training overhead, but this does not eliminate the costs of offline training, replay buffers, and multiple-network updates. Such methods are therefore most suitable when substantial training cost can be paid in advance, but online decisions must be produced quickly.

Wireless-FL studies further show that convergence and communication resources cannot be separated. User selection and uplink resource-block allocation directly affect model-transmission delay and convergence speed [134], while local-computation rounds, global-communication rounds, terminal energy consumption, and heterogeneous computing/power resources exhibit explicit trade-offs [135]. These studies provide general evidence for communication-learning coupling rather than SAGIN-specific performance claims.

### 7.2. Single-Agent DRL: Converting Dynamic Scheduling into State–Action–Reward Decisions

Fan et al. [29] consider a SAGIN containing both data-transmission and computation-intensive tasks. They construct a QoS-aware time-aggregated graph and priority queues and jointly model task scheduling, bandwidth allocation, and computing-resource allocation as a long-term dynamic decision problem. The state is not a complete topology matrix but consists of the task-state representation in Equation (28), covering node offloading and computing queues:

(28) $$s_{t}≜\left\{Q_{t}^{o},Q_{t}^{c}\right\}$$

The initial action includes task offloading *x_t_*, task processing *y_t_*, bandwidth allocation *b_t_*, and CPU-frequency allocation *f_t_*. To reduce dimensionality, offloading and bandwidth variables are further combined and uninformative actions are removed, compressing the action to $a_{t}≜x_{t}^{*},y_{t}^{*},f_{t}^{*}$ [29]. The reward exposes the optimization boundary directly through Equation (29):

(29) $$r_{t}≜-C(t)-N_{d}(t)$$

where *C*(*t*) is the system cost composed of latency and energy consumption, and *N_d_*(*t*) is the number of tasks dropped in the current slot. If only negative cost were used as reward, the agent could artificially reduce cost by processing fewer tasks. The authors therefore add an explicit drop penalty. This is an important design lesson: an RL reward should not be a mechanical negation of a mathematical cost function; it must also prevent policies from achieving superficially high return by violating service intent. The algorithm further uses generalized advantage estimation and a proximal policy optimization (PPO)-style clipped surrogate objective rather than a generic DQN formulation.

In civil-aviation simulations, the proposed policy stabilizes after approximately 900 episodes, whereas DDPG converges after roughly 700 episodes but with greater fluctuation. Across different task-latency tolerances, the proposed algorithm has a task-drop rate about 4.9% lower than DDPG; DDPG in turn outperforms the no-bandwidth-allocation and no-resource-allocation schemes by approximately 10.7% and 14.4%, respectively [29]. The authors also provide computational-complexity expressions related to neural-network input/output dimensions and identify broader task coverage and fast beam switching as future work. Single-agent DRL can therefore unify continuous-resource control with dynamic task queues, but expansion of state dimensions, action dimensions, and task types directly increases training complexity.

Zhang et al. [31] approach the scalability problem of centralized management in heterogeneous time-varying SAGIN by equipping each physical edge domain with an intelligent server capable of DRL. Each agent observes local CPU, storage, link bandwidth, and physical connectivity, i.e., $s_{i}(t)=\mathrm{CPU}(N_{i}^{P}),\mathrm{STO}(N_{i}^{P}),\mathrm{BW}(L_{i}^{P}),C_{i}(n^{P})$. During node mapping, CPU, storage, bandwidth, distance, and other features are used to construct a feature matrix. After successful mapping, the long-term revenue-to-cost ratio is used as reward, and the local policy network is updated by policy gradient. Agents optimize local link/node control while sharing part of their decision information to form a consistent solution [31].

The principal contribution of this work is its distributed control structure rather than DRL alone. Training is performed offline and testing online; BFS is used for online link allocation, resulting in an overall complexity of 𝒪(*N* + *E*) [31]. The abstract reports improvements of approximately 4.22% in long-term node utilization, 31.36% in link utilization, 11.75% in long-term average revenue-to-cost ratio, and 7.14% in acceptance rate. Detailed experiments report different gains against different baselines; for example, average node utilization exceeds GCN, SA, and MCRM by 48.52%, 26.47%, and 19.11%, respectively, while link utilization improves by 5.64%, 16.47%, and 13.15% [31]. These baseline-specific qualifications must be preserved rather than merging different percentages into one generalized claim. The authors also leave richer resource dimensions and deeper neural networks for future work, confirming that a distributed architecture does not eliminate all state-scale and model-capacity problems in large SAGIN.

### 7.3. Multi-Agent Reinforcement Learning: From Distributed Execution to Cooperative Training

When one centralized agent must simultaneously control many UAVs, users, and cross-layer resources, the action and observation spaces grow rapidly. Huynh et al. [30] therefore extend reliable task offloading from single-agent DDPG to cluster-based multi-agent DDPG (CMADDPG). The system maximizes the fraction of tasks that are successfully offloaded and executed within QoS deadlines while jointly selecting ground-user-to-UAV offloading ratios, ground-air bandwidth, and air-space bandwidth, subject to transmission-rate, total-bandwidth, energy-budget, and satellite-computing-capacity constraints.

For a single agent, the action can be written as *a_t_* = {*x_i_*_,*j*_(*t*), *b_i_*_,*j*_(*t*), *b_j_*(*t*)}, and the reward is the reliable-offloading ratio minus penalties for violating energy, bandwidth-budget, minimum-rate, and computing-capacity constraints [30]. In CMADDPG, each UAV acts as an independent agent for its own user cluster. During training, the critic can access the global joint state–action space, whereas during execution each actor acts only on local observations, implementing centralized training and distributed execution (CTDE). This structure addresses how local execution can preserve global coordination and is therefore stricter than simply running multiple independent DRL agents in parallel.

The original work also quantifies the cost. A single-agent DDPG update has complexity approximately *O*(*B*(*F_actor_* + *F_critic_*)), whereas MADDPG maintains M actor–critic pairs and requires each critic to process the joint state–action input of all agents, so computation grows approximately quadratically with M [30]. Simulations show that the single agent stabilizes after around 100 episodes with 15 users but around 250 episodes with 21 users. With 24 users, six UAV agents achieve better average reward and stability than three or four agents. For resource allocation, the optimized bandwidth scheme approaches a reliable-offloading ratio of one at lower computing load, and the proposed multi-agent method maintains lower average delay than Equal-Split and Random across different CPU demands [30]. The abstract further reports a reliable task-offloading rate up to approximately twice that of the baselines.

MARL is therefore not a cost-free solution in which more agents always improve scalability. More agents can reduce the local decision burden and support distributed execution, but joint critic inputs, experience sharing, and policy coordination increase training cost and memory use. Huynh et al. [30] do not yet include UAV trajectories as real-time decision variables and leave trajectory control and adaptive positioning to future work. In highly dynamic SAGIN, MARL design must jointly consider local observability, training scalability, communication overhead, and non-stationarity rather than comparing final average reward alone.

Beyond training complexity, the validity period of a learned policy is another deployment variable that is easily overlooked in dynamic SAGIN. Zhu et al. [27] organize model-aggregation routes by topology snapshots; Ref. [28] includes remaining satellite-service time in the HFL state; Ref. [31] uses offline training and online mapping; and ref. [30] leaves real-time UAV trajectory control for future work. Together, these settings show that when topology, service arrivals, or link statistics change on a timescale comparable to or faster than the model-update cycle, state–action relationships learned during training may already be partially stale at deployment. Low online inference latency therefore does not imply that a policy can be reused indefinitely. Evaluation should also report model/policy update periods, triggers for retraining or local re-optimization, and the effect of updating on service continuity. The notion of policy freshness used here is a survey-level synthesis from these evidence boundaries rather than a standardized metric already defined across the literature.

For scalability, training and execution placement should be co-designed with network hierarchy rather than assuming that all nodes carry equal learning load. HFL separates local training, LEO aggregation, and GEO management; distributed DRL pushes online decisions into physical domains; and CTDE keeps the expensive joint critic mainly in the training phase [27,30,31]. These structures correspond to different ways of shifting overhead: hierarchical aggregation of model traffic, local autonomy, and centralized training with distributed execution. They can reduce online pressure on particular nodes but convert it into model synchronization, cross-domain state sharing, centralized training, or multi-model maintenance. A distributed learning system should therefore be characterized not only by where control decisions are made, but also by where training data reside, where parameters are aggregated, where online inference runs, and which information still crosses domains.

### 7.4. Comparison of Learning Mechanisms and Section Boundaries

The preceding primary studies show that intelligent SAGIN resource optimization should not be reduced to three parallel labels—DRL, FL, and MARL. A more informative distinction is based on the learning object and decision structure. Zhu et al. [27] optimize model-aggregation traffic generated by hierarchical learning, with energy consumption, ISL topology, and reliability as the main tensions. Huang et al. [28] jointly optimize HFL performance and communication/mobility resources, with hybrid actions and fairness as the central difficulty. Fan et al. [29] use a single-agent PPO-style policy to jointly schedule tasks and communication/computing resources. Zhang et al. [31] push intelligent agents into multiple physical domains to reduce the centralized-control bottleneck. Huynh et al. [30] use CTDE to connect local execution by multiple UAVs with global cooperation. FL-VNFE [84] provides an additional example of FL for cross-domain SFC embedding, but its SFC service contribution has already been discussed in Section 5; only the local-training/global-aggregation learning mechanism is retained here.

From an engineering perspective, these methods expose four constraints that should not be hidden behind the phrase AI-enabled adaptation. First, training and execution are asymmetric. Zhang et al. [31] use offline training and online inference, and [28] also emphasizes that post-deployment prediction is much cheaper than training; fast online operation therefore does not imply low overall computational cost. Second, information sharing determines scalability. HFL requires multi-level model transmission, the centralized critic in MARL requires joint state–action information, and distributed DRL still shares part of the control information. Third, reward or aggregation objectives shape system behavior. Fan et al. [29] must add a task-drop penalty, while [28] dynamically regulates multi-task fairness. Fourth, topology mobility creates distribution shift for trained policies. Zhu et al. [27] explicitly exploit predictable topology and handle link interruption, whereas [30] still leaves real-time UAV trajectory control to future work.

Broader FL research further shows that non-IID data, device availability and system heterogeneity, communication efficiency, and privacy–robustness tension are not independent issues [136]. Hierarchical/federated learning in SAGIN should therefore be evaluated not only by model accuracy but also by communication, system, and security costs.

The deployability of learning-based SAGIN resource management can also be examined across its complete operational lifecycle. State/data acquisition determines observation quality; training or federated aggregation determines offline computing and communication cost; model/policy distribution determines update latency; online inference determines real-time control cost; and feedback/retraining determines whether the policy can track changes in topology and service distributions. Individual studies often emphasize only part of this chain: [27] focuses on aggregation communication, ref. [28] jointly controls model performance and multilayer connectivity, refs. [29,31] emphasize online decisions, and ref. [30] focuses on multi-agent cooperation. Horizontal comparison should therefore report training time/rounds, model-update traffic, online inference complexity, policy-update frequency, and performance degradation after state-distribution shifts in addition to final reward, accuracy, or task-completion rate. This prevents a method from being described as cost-free intelligence merely because computational burden has been shifted from online optimization to offline training.

Accordingly, this review treats intelligent learning as a decision and information-aggregation mechanism inside the resource-management control loop rather than as an algorithmic plug-in independent of the network model. Applicability should be evaluated along eight dimensions: state/observation size, action-space type, training mode, cooperation structure, online complexity, communication overhead, robustness, and generalization. Table 6 summarizes representative work using these dimensions.

This framework also establishes a direct methodological boundary for the smart-agriculture and smart-breeding applications in Section 9. Periodic sensing, UAV imagery, edge inference, task offloading, and digital-twin synchronization operate on different timescales. Whether learning is worthwhile therefore depends on whether relevant state is sufficiently observable, whether the online decision window permits inference, whether model-update traffic competes with service bandwidth, and whether policies generalize across seasons, fields, and devices. In other words, agricultural applications are not automatically better served by more complex DRL/MARL simply because they generate large datasets. Learning has practical deployment value only when it provides a clear long-term benefit over lower-complexity rules, heuristics, or auditable optimization after accounting for update cost.

Overall, learning-driven resource management is moving SAGIN from a model-then-offline-solve paradigm toward closed-loop operation based on continuous observation, online decision-making, and multi-node cooperation. Different learning paradigms, however, serve fundamentally different functions: FL/HFL enables model cooperation without centralized raw data, single-agent DRL handles high-dimensional continuous decisions, distributed DRL reduces centralized-control pressure, and MARL further coordinates multiple executors. The key future problem is not to stack increasingly complex neural networks, but to establish a unified evaluation framework for training cost, communication overhead, interpretability, cross-topology generalization, and security. On this basis, Section 8 turns to physical-layer security, privacy protection, trust, and security-aware orchestration.

## 8. Security Mechanisms and Privacy Protection

From the control-plane perspective, early SDN-security studies classified denial-of-service, trust, policy-conflict, and OpenFlow-related attack surfaces across the application, control, and data planes and their interfaces [137]. More recent work combining blockchain and DRL in next-generation wireless networks shows that trusted resource sharing and learning-driven caching/resource decisions can be jointly designed, but at the cost of consensus, signaling, and computation [138]. Security mechanisms should therefore be treated as endogenous constraints of resource-management objectives rather than as add-on modules applied after optimization.

At the authentication layer, the SAGIN-specific IMAS scheme uses satellite agents and multicast re-authentication to reduce authentication delay and signaling overhead during frequent handovers, but its public-key generation/trust center remains a single-point reliability risk [139]. The IMAS results indicate that lightweight authentication should be evaluated jointly in terms of security assurance, handover overhead, concentration of trust, and failure recovery.

For availability protection, LEO satellite swarms can exploit distributed receive diversity to improve anti-jamming resilience, and this capability has been validated through Universal Software Radio Peripheral (USRP)-based hardware-in-the-loop/over-the-air experiments [140]. For cross-domain attacks, a three-party game framework for satellite-enabled IoT jointly models attack propagation, cooperative defense, and incentive mismatch among IoT devices, terrestrial operators, and satellite service providers [141]. The former is physical-layer availability evidence, whereas the latter exposes security-efficiency trade-offs across operating domains; they cannot be represented by the same security metric.

**Figure 5:**
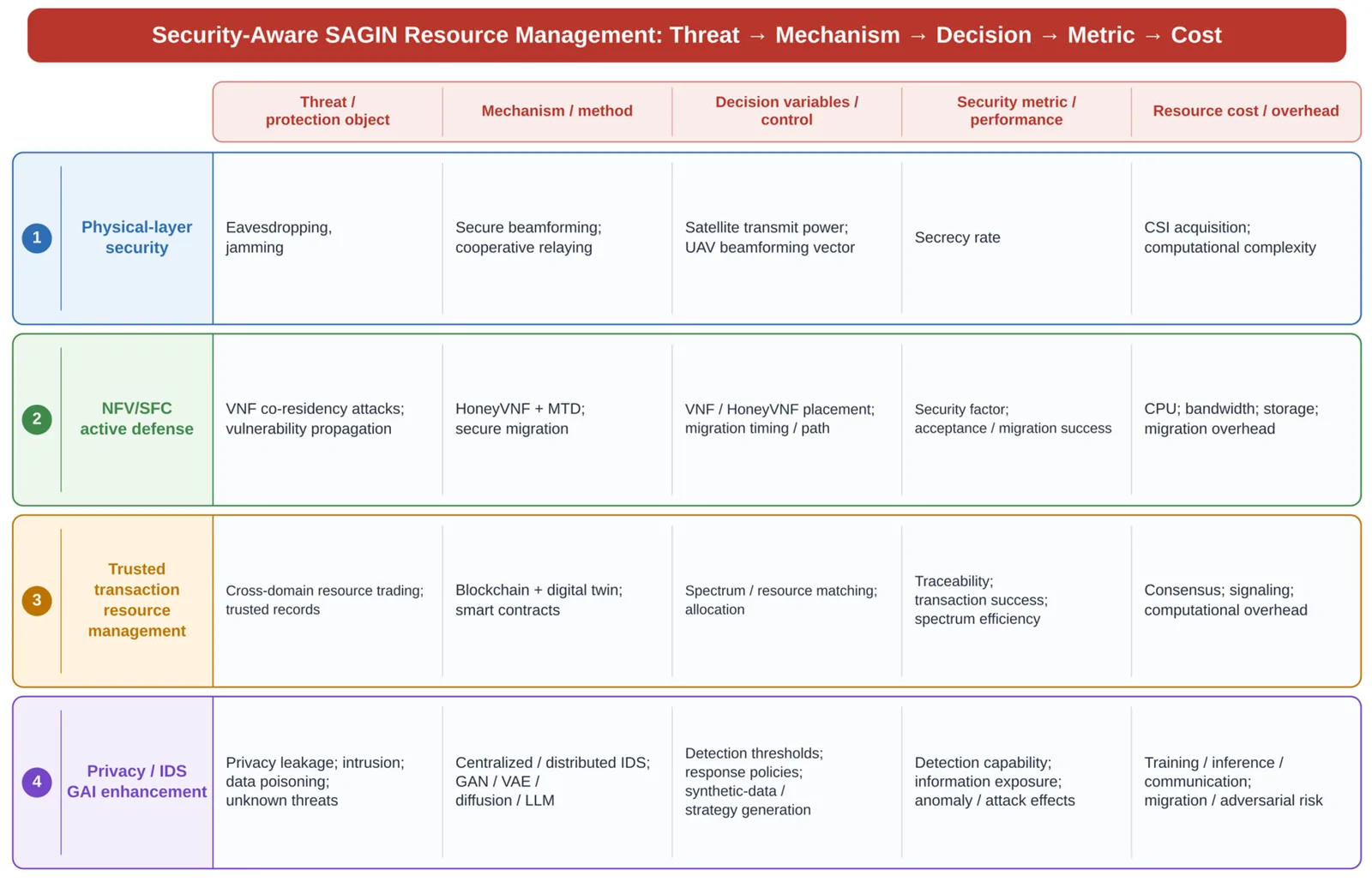
Taxonomy of security-aware SAGIN resource management from threat objects to resource costs.

Security in SAGIN is not a simple three-layer duplication of conventional wireless-security mechanisms across satellites, UAVs, and terrestrial networks. Cross-domain protocol heterogeneity, persistent topology changes, constrained onboard computation and energy, and the software attack surface introduced by SDN/NFV simultaneously affect authenticity, confidentiality, integrity, and availability. The SAGIN-security survey by Hu et al. [14] organizes threats across cross-domain interaction, space, air, and ground segments and further classifies attack vectors at the physical, data-link, and network layers, including eavesdropping and jamming, identity spoofing, malicious routing/packet tampering, resource exhaustion, and coordinated cross-domain attacks. This section therefore does not list encryption, blockchain, and AI as technology labels. Instead, it distinguishes four classes according to protection object and control location: physical-layer confidentiality, virtualization/SFC active defense, trusted transactions and auditing, and privacy protection with intelligent intrusion detection. Their security benefits and additional resource costs are analyzed separately. Broader physical-layer-security surveys for 5G and multi-user wireless networks [142,143], SDN-security work emphasizing centralized controllers, programmable interfaces, and data-plane attack surfaces [137], and differential-privacy surveys showing that FL does not automatically provide complete privacy merely because raw data remain local [144] are used as mechanism-level background. SAGIN-specific conclusions are grounded in the SAGIN studies discussed below. Figure 5 organizes the four classes along the chain threat object-mechanism-decision variable-security metric-resource cost.

In addition to network attacks, learning systems themselves face threats such as model extraction, model inversion/membership inference, adversarial examples, and data poisoning [145]. Using AI for security detection should not itself be treated as a new trust boundary.

More specifically, model updates exchanged during collaborative/federated training can leak membership information and task-irrelevant attributes of training data. Primary attack studies show that even when model averaging reduces exposure of individual gradients, it does not justify the conclusion that the training process has achieved complete privacy protection [146].

The optimization objective in a security section should not be compressed into one generic security score. Physical-layer methods directly optimize secrecy rate or eavesdropper suppression. NFV/SFC active defense jointly considers vulnerability/attack risk, security factors, service acceptance, load fairness, and migration cost. Trusted spectrum management focuses more on transaction traceability, spectrum utilization, and execution success. Privacy protection and intrusion detection must trade off attack-detection capability, information exposure, model overhead, and cross-domain deployment cost [14,32,33,34,35]. Each subsection therefore begins by specifying the protected object and attack model, then separates the security benefit from the associated resource cost, and only afterward discusses the algorithm or mechanism. This prevents direct comparison of fundamentally different security metrics.

From the resource-management perspective, the four security classes differ not only in attack target but also in where security state enters the control loop. Physical-layer confidentiality depends on channel or equivalent eavesdropping state, with an update rate constrained by channel variation. SFC active defense depends on VNF vulnerabilities, co-residency, satellite residence time, and service-chain state and is naturally tied to deployment/migration events. Trusted transactions require spectrum state, identity, and transaction history and are primarily limited by state consistency and execution latency. IDS/GAI requires continuous sampling, feature extraction, and model updating. A deployable security-aware resource manager therefore needs at least three timescales-fast link-level protection, event-triggered service migration, and slower trust/model updates-rather than assuming that all security states enter one optimizer at one common period. This multi-timescale view also explains why security gain must be evaluated jointly with state collection, signaling, computation, and execution latency [14,32,33,34,35].

### 8.1. Physical-Layer Security: Secrecy-Rate-Complexity Trade-Offs with Finite-Alphabet Inputs

Adjacent physical-layer-security studies cover artificial-noise-assisted secure beamforming, diversity-enhanced confidentiality, and secure transmission in heterogeneous cellular networks [147,148,149]. These provide mechanism-level baselines, but their channel, attacker, and topology assumptions are not equivalent to SAGIN. Their numerical performance is therefore not extrapolated directly to space–air–ground networks.

Physical-layer security exploits channel differences, power control, and beamforming to suppress eavesdroppers without relying on upper-layer protocols. Xia et al. [33] study downlink SAGIN in which a satellite transmits finite-alphabet symbols to a UAV swarm, the UAVs amplify and forward them to ground users, and a strong eavesdropper listens to both the satellite-to-UAV and UAV-to-ground stages. Unlike many secure-beamforming studies based on continuous Gaussian inputs, ref. [33] directly defines the achievable rates of the legitimate user and Eve using mutual information under discrete constellations such as quadrature amplitude modulation (QAM)/phase-shift keying (PSK) and expresses secrecy rate as $I_{s}(p_{s},w)={\left[I_{B}-I_{E}\right]}^{+}$. The core joint power/precoding problem in Equation (30) jointly chooses satellite transmit power *p_s_* and UAV precoding vector w to maximize *I_s_* under satellite-power and aggregate UAV forwarding-power constraints. Because finite-alphabet mutual information has no convenient closed form for direct optimization, the authors first construct a high-accuracy closed-form approximation *R_s_*(*p_s_*, w) using multi-exponential-decay fitting, with reported fitting errors generally below 10^−4^, and then apply alternating optimization: *p_s_* is optimized analytically for fixed w, followed by UAV-beam optimization for fixed *p_s_*.

(30) $$\underset{p_{s},w}{\max}R_{s}(p_{s},w),\;p_{s}\leq P_{s},\;(p_{s}|h_{u}{|}^{2}+\sigma_{u}^{2})w^{H}w\leq P_{u}$$

The beam-optimization stage provides two distinct engineering routes. CObI (convex-optimization-based iteration) converts the non-convex objective into iterative convex programs using lower-bound approximation and first-order linearization to preserve a high secrecy rate. HbSAI (heuristics-based semi-analytic iteration) replaces the objective with the ratio between the legitimate-user signal-to-noise ratio (SNR) and Eve SNR and exploits a generalized-eigenvalue structure for semi-analytic updates, substantially reducing solution complexity [33]. Simulation Figure 2, Figure 3 and Figure 4 show a clear performance-complexity trade-off. CObI and HbSAI both outperform MRT-OP for modulation orders M = 4, 16, 64. For M = 16, approximately 20 UAVs are sufficient for both methods to reach 95% of the maximum secrecy rate, whereas MRT-OP requires 36 UAVs. More importantly, HbSAI runs several orders of magnitude faster than CObI while maintaining a similar secrecy rate, which is attractive for computing-constrained UAV/onboard nodes [33]. The evidence boundary must be retained: the model assumes the channel knowledge and the specific two-hop downlink architecture defined in the paper, and its gains cannot be generalized to all SAGIN scenarios.

### 8.2. NFV/SFC Active Defense: From Vulnerability Quantification and HoneyVNFs to Secure Migration

NFV increases service-deployment flexibility, but co-locating multiple VNFs on shared satellites also expands side-channel, co-residency, and software-vulnerability propagation risks. Li et al. [34] do not treat security as an abstract weight; instead, they construct a three-level VNF-satellite-SFC risk model. For each VNF, the security factor in Equation (31) is *S*_vnf_ = 1 − *R*_vnf_, where attack probability is normalized from the vulnerability scores of co-resident VNFs. The vulnerability score itself combines CVSS-related confidentiality, integrity, and availability dimensions with side-channel exploitation probability. Satellite security is then derived from independent attack probabilities of hosted VNFs, while SFC security considers cascading risk across both constituent VNFs and their hosting satellites [34]. This is more rigorous than equating link reliability with security because attack objects and failure-propagation paths are explicitly separated.

(31) $$S_{{\mathrm{vnf}}_{i}}^{t}=1-R_{{\mathrm{vnf}}_{i}}^{t},\;S_{n_{m}}^{t}=1-R_{n_{m}}^{t}=\prod_{x_{p,m}^{t}=1}(1-R_{{\mathrm{vnf}}_{p}}^{t})$$

Two active mechanisms are introduced for defense. First, deception defense deploys HoneyVNFs that contain no sensitive data but expose system and port characteristics similar to real VNFs, redirecting attacker resources toward decoys and reducing exposure of real VNFs. Second, moving-target defense migrates affected VNFs and updates HoneyVNF security policies when a satellite-security factor drops below a threshold or topology changes threaten service continuity [34]. The secure-deployment problem P1, summarized in Equation (32), jointly maximizes SFC acceptance rate, average security revenue-to-cost ratio (ASR), and satellite-load fairness measured by the Jain index. PPO determines HoneyVNF locations and quantity from the current SFC/HoneyVNF deployment state, using a reward that directly combines security gain and load fairness rather than reusing the generic resource-scheduling rewards of Section 7. The migration problem jointly considers average migration cost, migration success, the change in ASR before and after migration, and load fairness. DS-SFC-M constructs a dynamic prediction factor from remaining satellite residence time, avoids placing VNFs on satellites about to leave the service area, and reconstructs only affected local chain segments where possible.

(32) $$P_{1}:\;\max\left(\alpha_{1}{\mathrm{succ}}^{t}+\alpha_{2}{\bar{r}}_{\sec}^{t}+\alpha_{3}f_{\mathrm{load}}^{t}\right)$$

Experiments use a 64-satellite Walker-star constellation with eight orbital planes. The results reveal the actual cost of active defense. HoneyVNFs approximately double the average SFC security factor relative to a no-security strategy, but their CPU consumption reduces the service-acceptance rate of SL-SFC by about 1–3% relative to LASO without security deployment. Compared with full-chain backup LVSCP, HoneyVNFs impose much lower security overhead. SL-SFC achieves the best load fairness and reduces CPU and bandwidth consumption by approximately 28% and 12.7%, respectively, relative to the compared schemes. When topology changes trigger migration, DS-SFC-M reduces the SFC migration ratio by at least 31% while maintaining a higher migration-success rate and more stable satellite/SFC security factors [34]. The most important conclusion is therefore not a single headline value such as 102% security improvement, but the measurable trade-off among security, acceptance rate, resource occupation, and migration frequency.

These results also show that SFC security cannot be achieved simply by adding a security weight to an ordinary placement objective. A practical closed loop contains at least six stages: risk observation, policy triggering, candidate migration-node screening, VNF/HoneyVNF reconfiguration, path updating, and post-migration risk verification. If risk estimation is too frequent, controllers and orchestrators incur excessive state-synchronization and reconfiguration overhead; if updates are too slow, VNFs can remain in high-risk co-resident environments. Secure migration therefore requires event thresholds, hysteresis, or minimum residence times to suppress oscillation and should compare expected risk reduction against CPU/bandwidth occupation, service-interruption time, and the probability of subsequent remigration. Li et al. [34] embody this logic through remaining residence time and local chain reconstruction, but the model parameters and attack probabilities are tied to a specific model, so cross-constellation and cross-service threshold design still requires validation.

### 8.3. Trusted Spectrum Transactions and Auditing: Blockchain Is Not Equivalent to Attack Resistance

Another class of security-related mechanisms operates at the resource-transaction and control layers. Liu and Zhao [35] propose a digital-twin-assisted blockchain framework for spectrum management. Their architecture contains physical, blockchain, and digital-twin layers. The physical layer collects spectrum/environmental states from satellites, UAVs, base stations, and users. The digital-twin layer constructs a dynamic virtual mapping through a knowledge graph and generates spectrum-sharing strategies. The blockchain layer treats physical nodes as on-chain participants and uses smart contracts to execute spectrum access automatically while recording transaction and usage histories. In this framework, blockchain provides decentralized recording, tamper resistance, traceability, and rule automation, whereas the digital twin compensates for blockchain’s limited ability to sense rapidly changing SAGIN states [35].

The concrete resource algorithm is not a cryptographic mechanism but a relational graph convolutional network (RGCN)+ComplEx-based spectrum–knowledge–graph completion. LEO satellites, satellite users, terrestrial base stations, terrestrial users, and frequency bands are modeled as entities; relations such as use and serve form triples; RGCN encoding and ComplEx decoding predict missing tail entities; and the final output is an available frequency band. The simulation includes one satellite, 50 base stations, 100 satellite users, 1000 terrestrial users, and 30 licensed bands. Transaction success is generally above 0.6, and spectrum efficiency consistently exceeds auction and random matching [35]. However, the paper does not experimentally construct forged transactions, 51% attacks, malicious smart contracts, or privacy leakage, nor does it report cryptographic security strength. It therefore supports only the claim that trusted recording/automatic execution can be coordinated with spectrum decisions, not that the framework has demonstrated resistance to network attacks. The authors also note that sparse knowledge graphs can bias completion and leave real-time performance and dynamic adaptability to future work.

At the control-plane implementation level, trusted transactions also face an often-overlooked final-consistency-versus-real-time trade-off. Spectrum or resource state is first collected from the physical network, mapped into a virtual representation by the digital twin, and then processed by the knowledge graph/smart contract to generate and execute an allocation. If topology, user association, or spectrum occupancy changes faster than twin updates and on-chain confirmation, the record may remain traceable but may not represent the freshest state at execution time. Trustworthiness therefore does not automatically mean real-time correctness. In addition to transaction success and spectrum efficiency, evaluation should report state age, twin-synchronization period, contract execution/confirmation latency, failed-transaction fraction, and rollback/conflict-resolution cost. Liu and Zhao [35] do not report these closed-loop indicators systematically, so the framework is positioned here as a prototype for trusted resource management rather than a complete solution to real-time consistency in highly dynamic SAGIN.

### 8.4. Privacy Protection and Intelligent Intrusion Detection: Evidence Levels and Deployment Location

Privacy protection and intrusion detection should be distinguished from the preceding active-control mechanisms. Hu et al. [14] summarize the core SAGIN-security requirements as authenticity, confidentiality, integrity, and availability and note that conventional AI in SAGIN is limited by scarce attack samples, non-IID data, difficulty in representing cross-domain features consistently, and frequent retraining. Their survey further discusses generative adversarial networks (GANs), variational autoencoders (VAEs), diffusion models, and large language models (LLMs) for security-data synthesis, cross-domain attack simulation, anomaly reconstruction, and security-policy generation, while explicitly identifying high compute/data requirements, adversarial examples and data poisoning, model inversion, LLM prompt attacks and hallucination, and domain-generalization risk under changing attack distributions. GAI is therefore treated here as a prospective security-intelligence enhancement layer rather than as evidence equivalent to the experimentally validated physical-layer algorithm in [33] or SFC active defense in [34].

For intrusion detection systems (IDSs), the strongest directly available source in this manuscript is the IoT IDS survey by Murali and Jamalipour [32], which is not SAGIN-specific and is therefore used only as generic support for mechanism classification and resource constraints. The survey classifies IDSs along three axes: host-based versus network-based data sources; signature, anomaly, specification, and hybrid detection logic; and centralized, distributed, or hybrid deployment. Centralized IDS can concentrate computationally intensive detection at powerful nodes but suffers from a centralized viewpoint and observability limitations during attacks. Distributed IDS can more readily detect internal attacks and support node cooperation but substantially increases energy, storage, and computation at each resource-constrained node [32]. These observations provide direct design implications for SAGIN, not direct SAGIN experimental results. Because onboard and aerial nodes have limited computing and energy budgets, deploying a full IDS at every node is generally unrealistic. A more plausible design layers lightweight feature extraction and local anomaly screening near space/aerial nodes with deeper analysis at terrestrial edge/cloud systems, using SDN global observability for cross-domain correlation. Accordingly, this is a design implication transferred from IoT resource constraints to SAGIN architecture rather than a basis for transplanting IoT accuracy values into SAGIN performance claims.

In generic edge/IoT systems, privacy-aware offloading has already shown that privacy level, computing latency, and terminal energy should be jointly optimized [150]. This result is not direct SAGIN performance evidence, but it supports the design principle that security constraints should be endogenous to resource-scheduling objectives.

For privacy and IDS, deployment location is itself a resource-management variable. Screening near satellite/UAV nodes can reduce further propagation of malicious traffic and limit raw-data exposure, but computing, storage, and energy constraints restrict such nodes to lightweight feature extraction, rule filtering, or low-complexity anomaly scoring. Centralized terrestrial edge/cloud deployment can run more complex models and aggregate cross-domain context, but increases uplink bandwidth, detection latency, and observability risk during disconnections. A more reasonable design is therefore not a binary choice between centralized and distributed IDS, but a hierarchical detection chain: resource-constrained nodes perform rapid screening and feature compression, edge nodes conduct regional correlation, terrestrial/cloud nodes perform deep analysis and model updates, and the SDN control plane translates alerts into isolation, rate limiting, path adjustment, or VNF migration. Since [32] provides only IoT deployment taxonomy and [14] mainly survey-level SAGIN security-intelligence mechanisms, this hierarchical loop remains a resource-constraint-driven architectural inference rather than a conclusion validated by a unified SAGIN experiment.

### 8.5. Comparison of Security Mechanisms and Resource-Management Boundaries

Security-aware SAGIN resource management contains at least four distinct optimization levels. Physical-layer confidentiality directly adjusts power and beams, using secrecy rate as a principal metric at the cost of channel-state dependence and solution complexity. NFV/SFC active defense adjusts VNF/HoneyVNF locations and migration policies and evaluates attack probability, security factors, service acceptance, and migration cost, while directly consuming CPU and bandwidth. Blockchain/digital-twin mechanisms address trusted cross-domain transactions and state recording, with benefits primarily in traceability, automatic execution, and resource-transaction success; security cannot be claimed independently of a concrete attack model. Intelligent IDS/GAI depends on data, models, and deployment location and introduces training, inference, communication, and model-security overhead while improving adaptability to unknown threats. Table 7 compares these mechanisms using threat object, decision variables, validation metrics, and principal costs.

SAGIN security mechanisms should not be compared only by whether they improve security. Evaluation should answer six questions simultaneously: What capabilities does the attacker possess? What object is protected? What information is observable? What are the decision variables? What security metric is validated? What resource cost is incurred? The existing primary studies show that secrecy rate, SFC security factors, migration success, transaction success, and IDS detection metrics are not interchangeable components of one universal security score. Future work should make security constraints endogenous to SDN/NFV orchestration: controllers should sense link and node risk, NFV orchestrators should assess co-residency and migration cost, and learning models should explicitly trade off cross-domain generalization, privacy, and adversarial robustness. Security gains should be reported jointly with CPU, bandwidth, energy, latency, service acceptance, and model overhead to avoid purchasing security through unacceptable service degradation. These security-resource trade-offs must ultimately be tested within concrete service chains.

The deployability of security-aware resource management can be summarized along four axes. Threat fidelity asks whether attacker capability, attack surface, and observation assumptions match the target scenario. Control coupling asks whether security state actually enters resource decisions involving power, paths, VNF locations, spectrum, or model deployment. Response lifecycle asks whether detection/triggering, decision, execution, verification, and rollback form a closed loop. Security cost captures communication, computing, storage, migration, training, consensus, and service-performance costs paid for the security gain. Evidence across all four axes is required to distinguish an effective security algorithm from a security mechanism that can operate sustainably within the resource budget of SAGIN.

For agricultural information and control, security mechanisms may protect phenotypic, environmental, and breeding-related data, digital-twin synchronization, and field-control commands while addressing requirements for privacy, integrity, and traceability. Any security gain should be evaluated jointly with latency, energy, bandwidth, resource occupancy, and service continuity. This is an architectural deployment implication: the Section 8 mechanisms provide a basis for mapping security requirements to SAGIN resource decisions, but do not constitute end-to-end agricultural validation. Section 9 therefore turns to smart agriculture and smart breeding as highly heterogeneous, long-horizon applications in which connectivity, computing, learning, and digital-twin requirements interact directly with SAGIN resource management.

## 9. Applications to Smart Agriculture and Smart Breeding

Smart agriculture uses digital technologies such as the Internet of Things, UAVs, wireless communications, cloud/edge computing, and artificial intelligence to connect environmental sensing, crop monitoring, equipment control, and production management into data-driven decision processes. Its objectives extend beyond improving production efficiency and crop yield to more precise use of water, fertilizer, energy, and other agricultural resources and to closed-loop management of time-sensitive events. Existing smart-agriculture surveys identify UAVs, wireless communications, SDN, NFV, and cloud/fog computing as important enabling technologies for agricultural IoT [151]. Smart breeding further extends this data-driven paradigm to long-term phenotyping, growth-process modeling, and cultivar decision-making. Agricultural networks must therefore carry not only periodic environmental measurements but also heterogeneous image, remote-sensing, phenotypic, and model-update data over long periods.

As sensing and intelligent devices continue to enter fields, greenhouses, and breeding sites, networking and computing requirements become increasingly heterogeneous. Agricultural production spaces are often geographically dispersed and unevenly served by infrastructure. Low-rate periodic monitoring, pest/disease or equipment-failure alarms, UAV image/phenotypic data, and control commands differ substantially in bandwidth, latency, reliability, and energy requirements. At the same time, edge and cloud nodes must share computing loads across real-time processing, data backhaul, and long-term model training. A single terrestrial access network or exclusive dependence on remote cloud processing is therefore unlikely to satisfy continuous connectivity in remote regions, mobile sensing, and latency-sensitive services simultaneously. These tensions motivate the following analysis of connectivity, task offloading, and digital twins.

**Figure 6:**
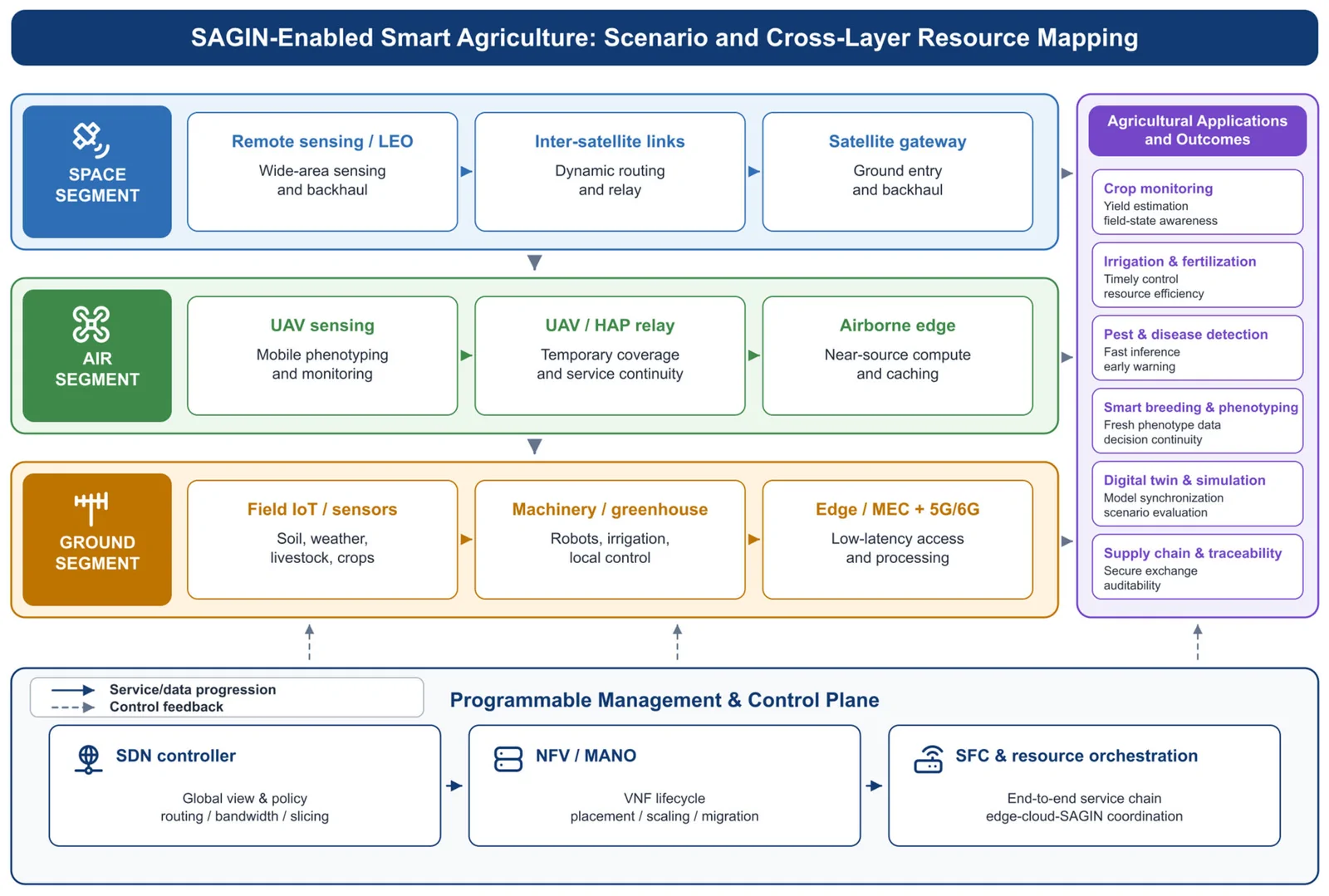
Overall smart-agriculture scenario and cross-layer resource-management mapping enabled by SAGIN.

In this context, SAGIN can use satellites for wide-area coverage and remote backhaul, UAVs/HAPs for mobile sensing, relaying, and temporary coverage, and terrestrial cellular, agricultural IoT, and edge-computing systems for local high-capacity access and real-time processing, forming a complementary multilayer agricultural information infrastructure [1,2,3]. The same heterogeneity, however, introduces changing coverage relationships, fluctuating link states, cross-domain resource differences, and service-priority conflicts. The value of SAGIN is therefore not merely the addition of more communication links. SDN-based global observability and programmable control, NFV-based service instantiation and migration, and learning-driven resource scheduling are needed to organize connectivity, control, computing, and digital-twin functions into an adaptive resource-management loop. On this basis, this section discusses ubiquitous connectivity and differentiated service assurance, edge computing and multi-resource orchestration, and digital twins and smart breeding, and then explicitly distinguishes agricultural evidence from SAGIN-oriented architectural inference. Figure 6 summarizes this cross-layer mapping.

### 9.1. Ubiquitous Connectivity and Differentiated Service Assurance

Remote agricultural scenarios are constrained by the economics, coverage radius, backhaul capability, and deployment cost of rural connectivity itself. A survey of rural connectivity notes that connecting the base of the pyramid requires scenario-specific combinations of satellite access, wireless access, and infrastructure cost [36]. SAGIN should therefore be viewed as an elastic complement to terrestrial agricultural networks rather than as an assumption that satellites or UAVs will necessarily replace existing rural communication systems.

Primary agricultural-monitoring studies further demonstrate the need for multilayer access. UAV-WSN systems have been used for precision-agriculture monitoring, while IoT-based precision-agriculture research in rural areas emphasizes sensing and connectivity under wide-area, low-infrastructure-density conditions [37,38]. This evidence is consistent with the application mapping adopted here, in which heterogeneous space–air–ground connectivity is assigned according to the agricultural task.

**Figure 7:**
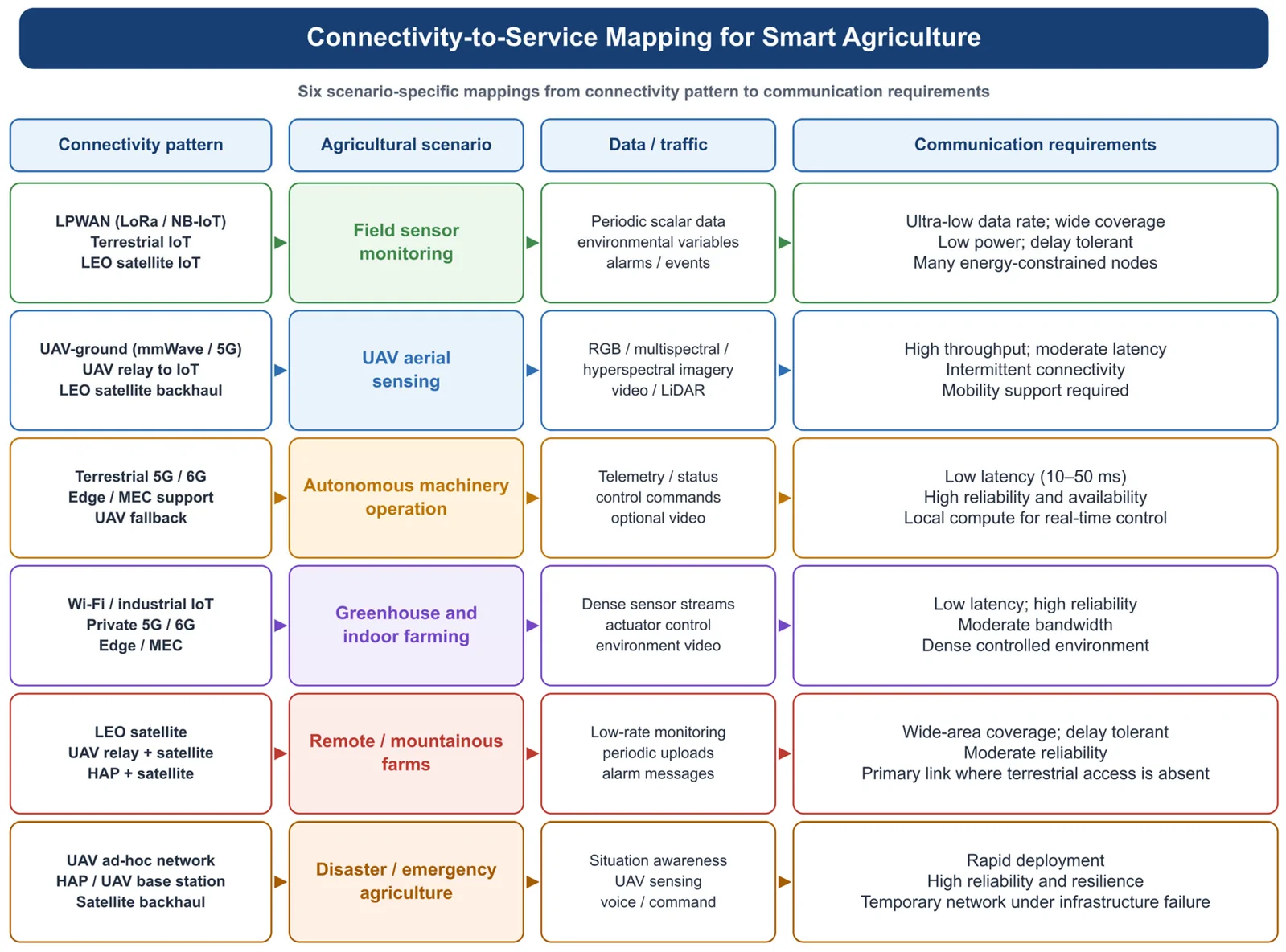
Connectivity types, application scenarios, and differentiated communication requirements in smart agriculture.

No single wireless technology can satisfy all agricultural services. A review of LoRa for Agriculture 4.0 shows that agricultural-IoT connectivity must jointly consider coverage distance, data rate, power consumption, propagation conditions in agricultural environments, relaying/routing, and sensor density, and compares the suitability of LoRa with Wi-Fi, radio-frequency identification (RFID), Bluetooth, and other technologies [39]. This evidence suggests that low-rate periodic sensing should prioritize low power and wide coverage, whereas UAV imagery, phenotypic images, and other data-intensive workloads require higher-throughput access or backhaul. For remote farms, last-mile access must also be considered together with the deployment cost of satellite or terrestrial backhaul. Ubiquitous connectivity in smart agriculture is therefore better implemented by combining short-range access, low-power wide-area networks (LPWANs), cellular networks, UAV relays, and satellite backhaul according to service type, while the control plane selects viable paths from service priority and real-time network state, rather than assigning one fixed technology to every agricultural task. Figure 7 summarizes these connectivity-to-service mappings.

Agricultural services exhibit pronounced spatiotemporal and QoS heterogeneity. Periodic environmental monitoring can generally tolerate some transmission delay, whereas pest/disease alarms, irrigation control, and equipment faults require greater timeliness and reliability. Masood et al. [40] classify smart-agriculture sensor traffic into emergency, normal, and on-demand classes using machine learning (ML), after which an SDN controller applies Q-learning for dynamic routing and bandwidth allocation according to traffic class and network state. The main value of SDN in this scheme is not centralized configuration alone, but the conversion of agricultural data semantics into programmable traffic priorities so that the control plane can adjust resource allocation to real-time network conditions. Random forest (RF) and decision-tree classifiers perform best on the reported dataset, and the combination of ML classification and Q-learning routing improves emergency-service delivery and latency [40]. The validation environment, however, remains predominantly a terrestrial IoT/SDN network and should not be interpreted as evidence that satellite–air–ground cross-domain handover has already been solved.

For coverage continuity in remote regions, AGRARIAN provides an agricultural architecture that is strongly analogous to layered SAGIN interconnection. The system combines IoT sensors, UAV monitoring, satellite connectivity, edge/cloud computing, and federated AI and organizes data flows into Sensor, Network, Data Processing, and Application layers [41]. Its network layer uses hybrid 5G-satellite backhaul, while its processing layer combines edge AI and eMBB slicing; pilot environments include vineyards and livestock farms [41]. This architecture suggests that satellites can compensate for wide-area or intermittent coverage, UAVs can serve as mobile sensing and edge nodes, and terrestrial edge/cloud systems can support deeper inference and long-term model training. AGRARIAN itself, however, does not validate a complete SDN/NFV-SAGIN control loop. It is therefore used here as application evidence for heterogeneous agricultural connectivity and hierarchical processing rather than as performance proof for a SAGIN resource-management algorithm.

### 9.2. Edge Computing, Task Offloading, and Multi-Resource Orchestration

From the broader perspective of precision-agriculture computing, the first value of edge computing lies in deciding whether data must leave the site at all. A recent review of edge computing for smart agriculture notes that when cloud connectivity is constrained in rural regions, moving part of the processing close to sensors and actuators can reduce real-time latency, lower backhaul consumption, and improve data locality and security. Edge solutions should therefore be evaluated not only by algorithmic accuracy but also by deployment metrics such as latency, accuracy, memory footprint, and power consumption [42]. This framework maps directly onto SAGIN resource management: latency-sensitive control and lightweight inference are better suited to field gateways, UAVs, or terrestrial MEC, whereas large-scale model training, cross-season analysis, and digital-twin knowledge updates may remain at regional edge or cloud systems. When satellite or UAV links participate in backhaul, processing placement is further constrained by visibility windows, link capacity, energy consumption, and task deadlines.

At the implementation level, smart-farming platforms have already used hierarchical edge/cloud architectures for greenhouse sensing, control, and data processing, providing direct application evidence for offloading cloud analytics functions and virtualized resource management [43]. Other edge/IoT platforms integrate sensing, edge computing, and AI with agricultural production-chain monitoring for real-time management of livestock and crop conditions [44]. These deployments connect the edge-orchestration discussion in this section to actual hierarchical processing requirements in agricultural systems rather than leaving it as a SAGIN-only architectural projection.

**Figure 8:**
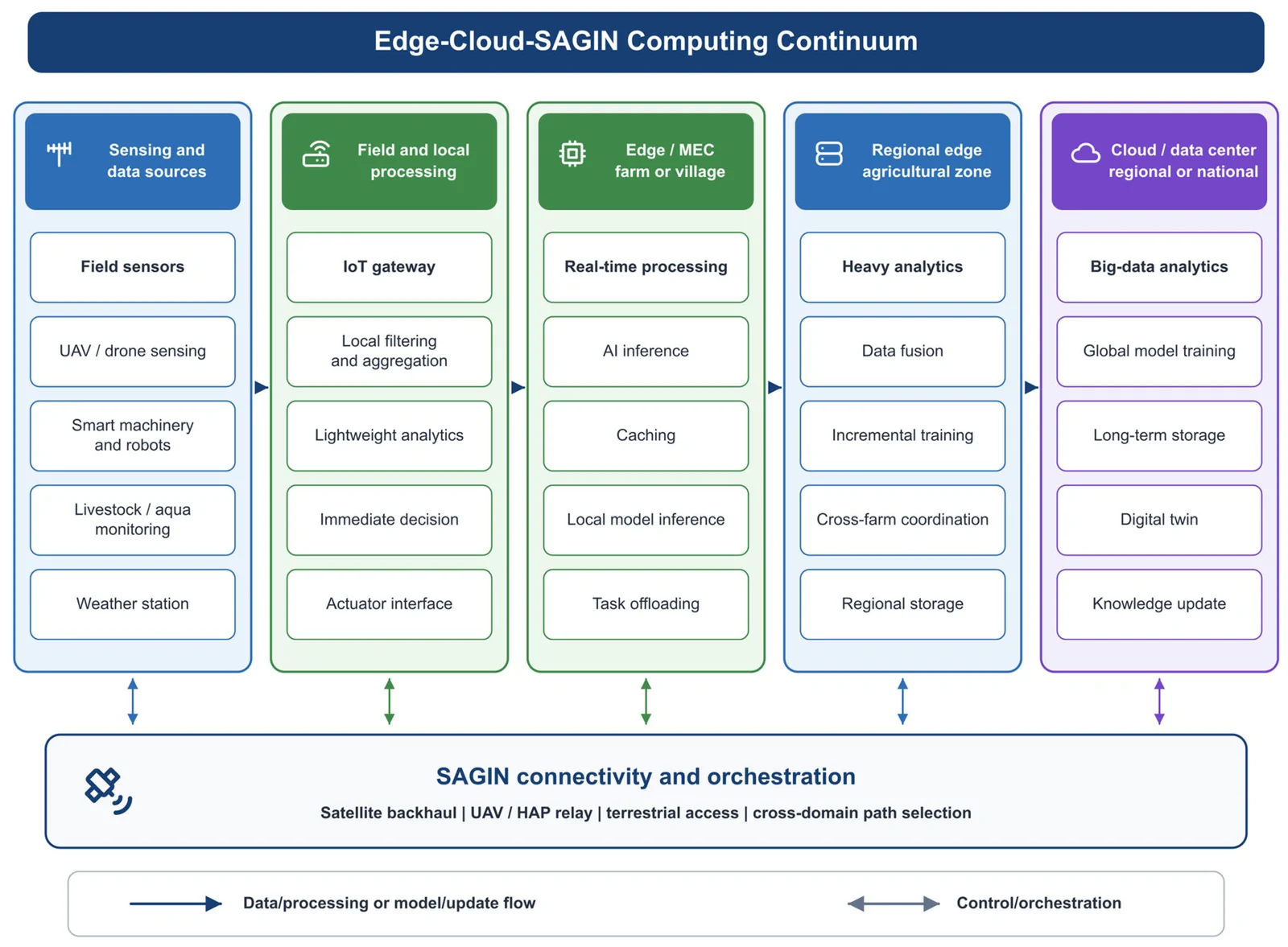
Edge/Cloud-SAGIN computing continuum and data-processing chain for smart agriculture.

As agricultural endpoints expand from low-rate sensors to cameras, UAVs, and multimodal sensing devices, the bottleneck shifts from link bandwidth alone to joint contention for communication, computing, and caching resources. Zhao et al. [45] consider smart-agriculture scenarios in which multiple tasks reuse the same images or sensor data and jointly formulate task offloading and resource orchestration to minimize latency, energy consumption, and cache occupancy under communication, computing, and caching constraints. They develop the hierarchical-reward multi-agent DRL algorithm MATD3-CO-RO-MDS. The work emphasizes that agricultural tasks differ in data volume, latency, and energy demand and that repeated transmission of shared data wastes both communication and caching resources. As the number of offloaded tasks increases, the proposed scheme achieves up to a 76.7% latency-reduction advantage over the compared algorithms [45]. This result supports the need for cross-resource orchestration when multiple services share data, but the system remains an agricultural MEC environment and does not include SAGIN-specific dynamics such as LEO topology, ISLs, or cross-domain controllers. Figure 8 summarizes the resulting edge–cloud–SAGIN computing continuum.

GSAgri reveals a complementary green and security-aware perspective on agricultural resource management. It constructs a cooperative MEC/edge/cloud system with energy-harvesting IoT devices and combines simulated-annealing-style task offloading, heuristic scheduling, and gated recurrent unit (GRU)-based solar-energy prediction to jointly reduce application latency and terminal energy while incorporating critical-task and security requirements into offloading decisions [46]. Across simulation, a real agricultural disease-prediction application, and real network data, the study reports substantial gains, including reductions of 19.19% in average application latency and 62.76% in device energy consumption relative to the compared scheme, with a 16.57% latency improvement under real network data [46]. The authors also note that prioritizing critical tasks can increase waiting time for non-critical tasks and cause deadline violations. Timeliness, energy, criticality, and fairness therefore cannot be compressed into one optimality metric. This trade-off directly parallels the energy budgets, link availability, and service-priority constraints of spaceborne and aerial nodes in SAGIN.

### 9.3. Digital Twins, Phenotyping, and Smart Breeding

From an agronomic-management perspective, the core of an agricultural digital twin is not a static three-dimensional model, but a virtual representation that continuously assimilates physical-system state at a defined frequency and accuracy and feeds predictions or optimization results back into management. Reviews of digital twins in agriculture discuss applications across soil/field conditions, crop management, postharvest processes, and farm infrastructure and identify irrigation, fertilization, pest/disease management, and decision support as representative scenarios. They also identify managing large amounts of data and multiple, often simultaneous agricultural operations as a major challenge [47]. The networking value considered in this subsection should therefore be understood through the joint constraints of data freshness, synchronization frequency, processing location, and decision timeliness rather than reducing a digital twin to cloud visualization.

**Figure 9:**
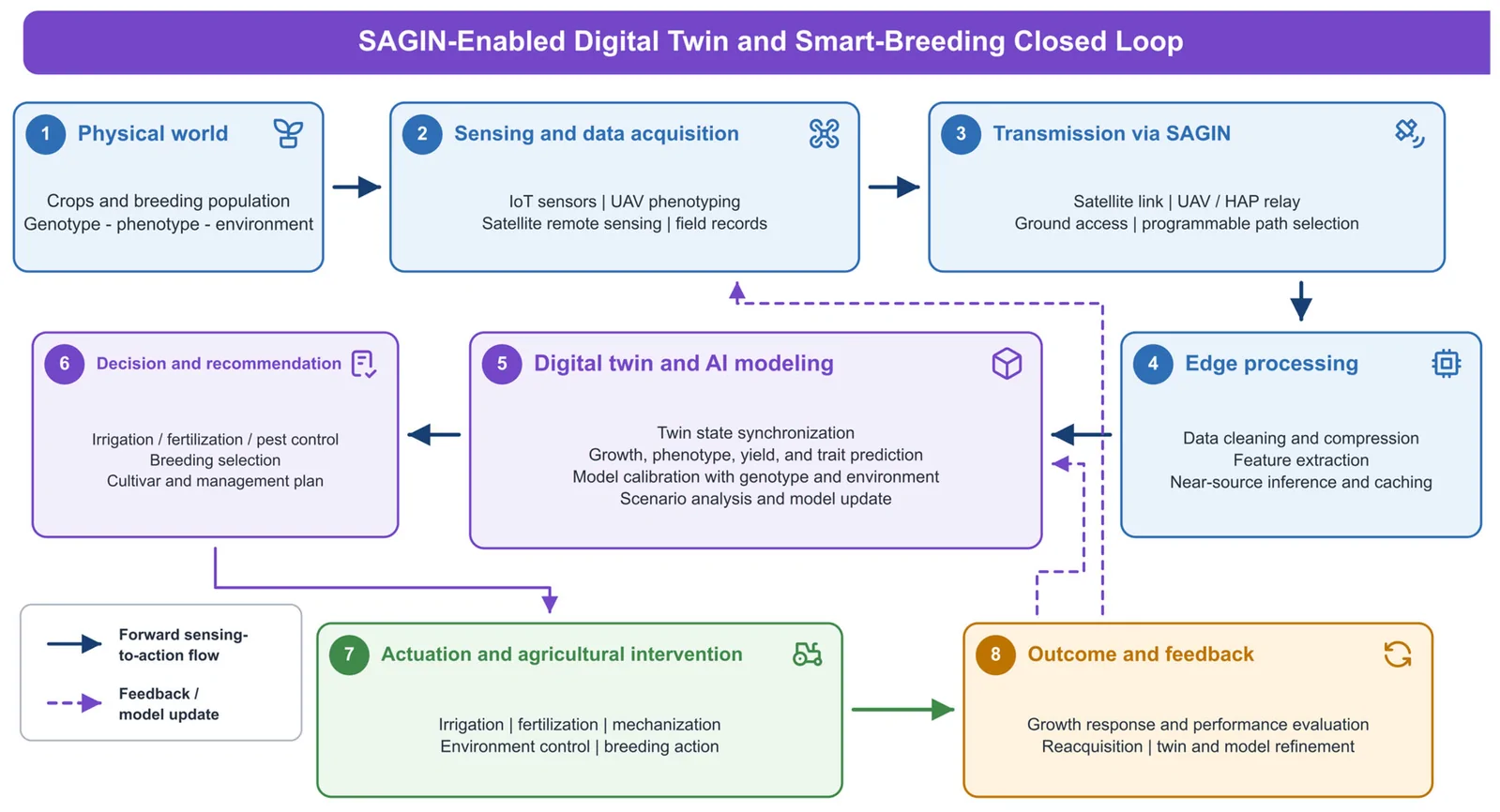
SAGIN-enabled digital-twin and smart-breeding closed loop.

At the systems level, Awais et al. [48] organize IoT sensors, UAVs, remote sensing, machine learning, and real-time data integration into a digital-twin framework in a systematic review of studies from 2018 to 2025. A review of unmanned aircraft system (UAS)-based high-throughput phenotyping (HTP) for plant breeding further identifies the labor and time cost of manual phenotyping as a major breeding bottleneck and shows that UAS-HTP can support direct trait measurement, predictive breeding, and marker/quantitative trait locus (QTL)-related analysis to improve genetic gain [49]. The two reviews show that smart breeding needs more than the ability to transmit images. It requires stable organization of high-throughput phenotypic data across time and fields and persistent alignment of these data with environmental conditions, cultivar/genotype context, and model updates. Figure 9 illustrates this closed-loop organization.

Smart breeding differs from general smart agriculture in that the system must not only optimize irrigation, fertilization, or equipment scheduling, but also maintain relationships among genotype, phenotype, and environment over multiple growth cycles and support predictive and cultivar-level decisions. Liu et al. [50] combine non-destructive plant phenotyping with functional-structural plant modeling and use real-time phenotypic data to calibrate growth models, forming a dynamic plant digital twin capable of predicting the full growth process. They note that conventional manual phenotyping is time-consuming, expensive, and destructive and therefore difficult to scale to large breeding populations; image/3D reconstruction and deep learning can improve phenotyping efficiency across growth stages [50]. Their discussion further links phenotypic traits to genotype and environmental factors and applies digital twins to growth prediction, environmental-adaptation analysis, and breeding decisions. From the breeding perspective, the key networking requirement is therefore not one-time data upload, but a long-term traceable loop of phenotyping, model synchronization, prediction, and reacquisition.

The winter-wheat digital twin of Skobelev et al. [51] further incorporates cultivar explicitly. Its multiparametric variety model combines crop ontologies, a knowledge base, and a multi-agent digital-twin structure and jointly uses genetic characteristics, environmental factors, phenological stages, and resource conditions to predict yield and quality parameters and to support decisions on cultivar, sowing date, fertilization, and crop protection. Initial validation uses actual field data from 2023, but the authors report that some parameter predictions remain insufficiently accurate and plan to retrain satisfaction functions using subsequent measurements [51]. This is an important caution for smart-breeding reviews: a more complex digital twin is not automatically a more accurate one. Effectiveness depends on cross-season data quality, cultivar-parameter calibration, and synchronization with the real environment.

### 9.4. SAGIN-Oriented Application Mapping and Research Boundaries

The agricultural evidence above supports an application mapping for SAGIN. Terrestrial sensors and breeding platforms perform continuous environmental and phenotypic sensing; UAVs provide mobile imaging and local relaying; satellites provide wide-area connectivity and complementary remote sensing in remote regions; edge nodes support data filtering, lightweight inference, and latency-sensitive tasks; and cloud systems handle long-term model training and digital-twin knowledge updates. The SDN control plane can adjust routing and bandwidth according to service priority, link state, and coverage changes, while NFV has the potential to deploy functions such as phenotype preprocessing, disease recognition, data fusion, and twin synchronization as migratable service chains across heterogeneous nodes. The evidence boundary is important: existing literature directly supports agricultural SDN traffic control, hybrid satellite/UAV connectivity, MEC offloading, and digital-twin-assisted breeding, whereas direct evidence integrating SDN/NFV-enabled SAGIN resource management with end-to-end agricultural or smart-breeding deployments remains limited in the literature covered by this review. NFV/SFC deployment for smart breeding is therefore an architectural inference derived from the mechanisms in Section 5 and Section 6 and agricultural service requirements, not an experimentally demonstrated agricultural system.

Representative deployed or field-grounded cases further anchor this mapping. AGRARIAN integrates IoT sensors, UAV monitoring, hybrid 5G-satellite backhaul, edge/cloud processing, and federated AI in pilot environments including vineyards and livestock farms [41]. Hierarchical edge/cloud and edge/IoT platforms have also been deployed for greenhouse sensing/control and livestock/crop monitoring [43,44]. GSAgri evaluates a real agricultural disease-prediction application and real network data, showing latency- and energy-related benefits while also exposing deadline/fairness trade-offs [46]. For smart breeding, the winter-wheat digital twin in [51] is initially validated using actual field data from 2023. These cases demonstrate real agricultural connectivity, edge-processing, and digital-twin requirements, but they should not be interpreted as experimental validation of a complete end-to-end SDN+NFV+SAGIN architecture.

These workloads can be translated into application-level service requirements that play an SLA-like role in orchestration. Periodic environmental monitoring prioritizes energy efficiency, coverage continuity, and delivery availability; pest/disease alarms and irrigation/equipment control require tighter delay and reliability bounds; UAV imagery and phenotyping require burst bandwidth, data freshness, and edge-computing capacity; and digital-twin/breeding synchronization requires long-horizon continuity, update consistency, and traceability. The SDN control plane can translate these requirements into routing, bandwidth, priority, and admission policies, whereas NFV/SFC orchestration can translate them into VNF placement, scaling, migration, and service-chain decisions. When multiple agricultural services contend for UAV, edge, communication, and computing resources, feasibility should therefore be judged against service-specific requirements rather than a single network-wide QoS target.

Improved QoS is not resource-neutral. Lower latency can require additional edge capacity or more aggressive placement; higher reliability may require redundant paths or reserved resources; fresher digital-twin state increases synchronization traffic and computing load; and VNF migration can preserve service continuity at the cost of state-transfer traffic, reconfiguration delay, and energy. Consistent with the trade-offs reviewed in Section 4, Section 5 and Section 6, agricultural evaluations should report service-quality gains together with communication, computing, energy, and reconfiguration costs. This prevents an apparent QoS improvement from being interpreted as a net agricultural benefit when it merely shifts cost to constrained UAVs, edge nodes, or backhaul links.

These mappings also expose four unresolved deployment questions. First, connectivity interruptions, satellite/UAV coverage changes, and edge-node load changes should be jointly modeled with phenotyping/digital-twin synchronization periods; otherwise, twins may continue using stale data after network conditions change. Second, agricultural task priority should not be determined only by emergency/normal labels, but should also consider breeding stage, data irrecoverability, model-update value, and terminal-energy budgets. Third, geographically distributed breeding introduces heterogeneity across phenotypic, environmental, genetic, and management data, requiring orchestration to consider data sovereignty, privacy, and traceability. Fourth, current agricultural studies often optimize routing, MEC offloading, or digital-twin models separately and rarely report communication latency, computing energy, cache occupancy, model-prediction error, and breeding-decision benefit within the same real experiment. Together, these characteristics make smart agriculture and smart breeding heterogeneous, long-horizon closed-loop scenarios in which the deployability of cross-domain SDN/NFV resource management can be evaluated.

## 10. Future Research Directions and Challenges

Building on the preceding analysis of controller placement, SFC orchestration, SDN/NFV cooperation, learning-driven resource management, and security mechanisms, and on the smart-agriculture/smart-breeding mapping in Section 9, future research should not be summarized simply as using more complex AI or deploying larger constellations. The agricultural relevance of the six directions below should be explicit: onboard and hierarchical intelligence should preserve edge-inference timeliness and model freshness; integrated communication–sensing–computing should improve sensing and phenotyping quality without violating control deadlines; digital-twin autonomy should reduce synchronization error and decision staleness; green resource management should extend monitoring and UAV service continuity under energy limits; distributed LEO management should maintain remote-site connectivity as network scale increases; and security mechanisms should protect agricultural data and control commands without unacceptable latency or resource overhead. Accordingly, network metrics should be linked to agricultural-task outcomes such as phenotypic-data freshness, alarm/control response deadlines, availability of edge inference, cross-season model synchronization, and data integrity required for breeding and management decisions. The first five directions are directly supported by existing SAGIN or closely related evidence, whereas post-quantum and quantum-link security is presented as a longer-term interdisciplinary direction and remains clearly separated from the experimental evidence reviewed above.

### 10.1. Onboard Intelligence and Hierarchical Learning

Section 7 shows that the bottlenecks of hierarchical/federated learning in SAGIN arise not only from model training but also from model-update traffic, aggregation topology, link interruption, and non-IID data. Zhu et al. [27] explicitly formulate model-aggregation routing as a topology-optimization problem involving energy and reliability, while [28] further includes UAV trajectories, remaining satellite-service time, user/satellite association, and multi-level aggregation weights in HFL control. Future work should therefore prioritize lightweight models, asynchronous/event-triggered aggregation, cross-orbit model routing, and joint training–communication–energy budgets rather than assuming that onboard nodes can sustain data-center-scale training. On the security side, the GAI survey [14] also highlights the need for model compression and distributed training/inference in resource-constrained SAGIN, together with risks from data poisoning, model inversion, and adversarial attacks.

### 10.2. Cross-Layer Cooperation for Integrated Communication, Sensing, and Computing

SAGIN research is extending from single communication-resource optimization toward joint communication, sensing, and computing. Zhang et al. [152] present an architecture and handover procedure for joint communication, sensing, and computing in SAGIN. The open resource-management problem is not to maximize all three functions independently, but to construct unified state representations and characterize interpretable trade-offs among waveform/spectrum use, sensing accuracy, computing-offload latency, and energy. Future work should study Pareto frontiers under multi-timescale constraints, task priorities, and imperfect state information and clarify which variables are coordinated by the SDN control plane and which are executed by NFV/edge-computing systems, thereby avoiding an architectural treatment of integrated communication–sensing–computing without operational resource decisions. Joint communication–sensing–computing in satellite IoT has likewise been identified as a key direction for resource reuse and cross-function cooperation [153]. At the methodological level, learning algorithms for integrated sensing and communication (ISAC) have been systematically studied for resource management, waveform design, and wireless sensing [154]. For SAGIN, the more valuable question is therefore how a learning agent produces interpretable cross-layer decisions under shared spectrum, sensing tasks, and computing budgets rather than simply adding an AI module.

### 10.3. Digital-Twin-Driven Closed-Loop Autonomy

Liu and Zhao [35] provide an initial loop of physical-state acquisition, digital-twin/knowledge-graph modeling, policy generation, smart-contract execution, and feedback updating, but validation focuses on spectrum utilization and transaction success rather than synchronization accuracy and robustness of large-scale autonomous networking. Future digital-twin research should explicitly include model error, state-synchronization period, prediction mismatch, and control-action risk within resource-management objectives and establish an auditable loop from twin recommendation to real-network execution and deviation correction. In rapidly changing LEO topologies, if twin updates are slower than network-state changes, decisions based on stale states may become invalid. Multi-fidelity twins, event-triggered synchronization, and sim-to-real policy transfer therefore deserve further investigation.

### 10.4. Green and Sustainable Resource Management

The analyses in Section 6 and Section 7 show that energy consumption should not be treated merely as a post hoc statistic. Zhu et al. [27] directly optimize the energy consumed by model-aggregation routing, while [28] jointly controls hierarchical learning and communication resources. Future green SAGIN should place communication energy, computing energy, model-training/inference energy, and migration/reconfiguration overhead within one budget while jointly constraining QoS, reliability, and model performance. Relevant directions include low-complexity online policies, on-demand sleep/wake operation, energy-aware VNF migration and model aggregation, and robust scheduling under uncertain renewable-energy availability. Evaluation should extend from a single energy-saving percentage to energy per completed service, per unit improvement in model accuracy, or per unit of reliable service. Edge AI itself also incurs substantial energy cost: large-scale data acquisition and deep neural network (DNN) training/inference consume energy on constrained edge devices, and green-edge-AI research therefore treats the energy efficiency of data acquisition, edge training, and edge inference as one continuous design chain [155]. This perspective broadens the evaluation boundary of green SAGIN beyond communication or migration energy to include the energy cost of the learning model itself.

### 10.5. Dynamic and Distributed Management for Very Large LEO Constellations

Existing work already treats dynamic topology, link lifetime, and cross-domain state updates as major SAGIN resource-management constraints, but the evidence reviewed here is insufficient to support absolute predictions such as an inevitable increase from thousands to tens of thousands of satellites or exponential growth in beam-management complexity. A more defensible research question is how the communication and computational overhead of centralized global-state collection scales as node count, visibility relationships, and handover events increase. Zhang et al. [31] use distributed DRL to reduce centralized-control pressure; [30] shows that the computational burden of a multi-agent critic grows substantially with agent count; and [34] uses remaining satellite connection time to reduce dynamic SFC migration. Future work should therefore investigate hierarchical control, local autonomy, event-triggered state sharing, topology prediction, and consistency across control domains so that algorithmic complexity and signaling scale controllably with constellation size rather than assuming that one central controller continuously maintains a complete real-time view.

At the constellation scale, ultra-dense LEO constellation design has examined large satellite deployments and terrestrial/satellite integration from the perspective of global coverage [156]. This review does not infer a fixed future satellite count from that literature, but uses it as background evidence that increasing node scale amplifies association, handover, state synchronization, and control overhead.

### 10.6. Post-Quantum and Quantum-Link Security as a Long-Term Interdisciplinary Direction

The SAGIN-security work reviewed in Section 8 primarily covers physical-layer confidentiality, SFC active defense, trusted transactions, and intelligent security analysis. SAGIN-specific experiments on post-quantum cryptography or quantum key distribution remain limited in the evidence base of this review. This direction is therefore presented only as a research outlook, not as evidence that current technology is mature or that conventional cryptography is about to fail. Key questions include the key-size, computational, and latency overhead of post-quantum algorithms on resource-constrained spaceborne/aerial nodes; cooperation between key-update mechanisms and SDN control processes under long-delay, intermittent connectivity; and whether quantum/post-quantum mechanisms materially alter the resource budgets of SFC deployment, controller load, and service QoS. Stronger conclusions require more SAGIN-specific primary experiments that establish an explicit security-performance-versus-resource-cost evidence chain.

These six directions share one central problem: future SAGIN resource management should no longer optimize network performance, learning performance, security, and resource overhead in isolation. More verifiable research should organize dynamic state sensing, cross-layer decision-making, online execution, and feedback correction into closed loops and report gains and costs in the same experiment, for example, latency/acceptance versus signaling overhead, model accuracy versus training energy, security gain versus resource occupancy, and autonomy versus decision risk. For smart agriculture and smart breeding, the same evaluation should also report agricultural outcomes, including alarm/control timeliness, phenotypic-data completeness and freshness, edge-inference availability, digital-twin synchronization error, and continuity of breeding or management decisions. Only under such joint evaluation can algorithms be compared fairly at the level of deployability.

## 11. Conclusions

Motivated by the digital-infrastructure requirements of smart agriculture and smart breeding, this review systematically examines progress in SDN- and NFV-enabled SAGIN resource management. Across SDN controller placement, NFV/SFC orchestration, SDN/NFV cooperation, learning-driven resource management, and security mechanisms, primary evidence is compared in terms of optimization objectives, decision variables, solution mechanisms, application scenarios, quantitative gains, and engineering costs. Smart agriculture and smart breeding are used to test the practical requirements of cross-domain resource management for connectivity, computing, task offloading, phenotypic sensing, and digital twins. The literature shows a clear transition from static, single-domain, single-objective optimization toward closed-loop management that combines dynamic cross-domain state sensing, service orchestration, and learning-based decisions. For agricultural applications, the value of this transition lies in organizing remote coverage, mobile sensing, edge processing, and long-term model synchronization within one schedulable infrastructure rather than simply adding another communication link. Explicit trade-offs nevertheless remain between controller count and control latency, SFC benefit and migration cost, learning performance and communication/computing overhead, and security gain and service acceptance.

From an engineering/deployability perspective, current methods face four major boundaries. First, highly dynamic topology makes centralized optimization based on complete real-time global state vulnerable to stale information and signaling overhead. Second, onboard and aerial processing, storage, and energy budgets constrain complex online search and large-model training. Third, many learning-based approaches still depend on specific simulation topologies, traffic models, and state-observability assumptions and generalize poorly across constellations, services, and failure conditions. Fourth, security mechanisms consume CPU, bandwidth, and energy or introduce migration/verification overhead and therefore cannot be evaluated independently of the resource budget. This review also has a defined scope: it focuses on SDN/NFV-enabled resource management rather than exhaustively covering physical-layer transmission, control-plane protocol implementation, every security attack surface, or quantum/post-quantum experimentation. Generic wireless, IoT, and edge-computing literature is used only as mechanism-level background and design guidance and is explicitly distinguished from SAGIN-specific experimental conclusions.

For practical systems, a more robust design path is hierarchical control, scenario-specific modeling, and multi-timescale adaptation rather than pursuit of one global optimum. Relatively stable baseline deployment and capacity planning can be handled by auditable mathematical models; topology and load changes can be addressed by low-overhead heuristics, local migration, or event-triggered mechanisms; and DRL/MARL or federated/hierarchical learning can be introduced when state and action spaces become sufficiently complex. Reliability, security, energy consumption, and control/training overhead should be reported as components of the same resource budget. For smart agriculture and smart breeding, evaluations should further report how these networking and computing costs affect the agricultural task itself, including alarm/control timeliness, completeness and freshness of phenotypic data, continuity of edge analytics, digital-twin synchronization error, and the availability of decisions across growth cycles. Onboard intelligence and hierarchical learning, integrated communication–sensing–computing, digital-twin closed-loop autonomy, green resource management, distributed management for very large LEO constellations, and post-quantum/quantum-link security remain important directions. The first five already have direct or adjacent SAGIN evidence, whereas post-quantum security still requires more SAGIN-specific primary experiments. Only when experiments jointly report network performance, resource cost, robustness, security boundaries, and outcomes relevant to the target agricultural task can SDN/NFV-enabled SAGIN solutions be meaningfully compared, reproduced, and deployed in digital-agriculture scenarios.

## Figures and Tables

**Table 1:** Comparison of prior research directions and the gap addressed by this review.

Research Direction	Representative Refs	Main Focus	Limitation Relative to This Review	Agriculture/SDN-NFV Relevance
SAGIN architecture and channel modeling	[1,2,15]	Architecture, coverage, channel/system performance	Upper-layer programmable control and service orchestration are not the main focus	Agriculture indirect; SDN/NFV limited
SDN controller placement	[16,17,18,19]	Control latency, load balance, controller placement/association	Limited coupling with VNF/SFC lifecycle decisions	SDN direct; agriculture indirect
NFV/SFC orchestration	[20,21,22,23]	VNF placement, migration, chaining, latency/cost	Control-plane cooperation is only partly represented	NFV/SFC direct; agriculture indirect
Cooperative SDN/NFV management	[21,24,25,26]	Joint state, virtual-resource embedding, closed-loop orchestration	Agriculture-oriented cross-layer evaluation remains limited	SDN/NFV direct; agriculture indirect
Learning- and security-aware SAGIN	[14,27,28,29,30,31,32,33,34,35]	Learning-based allocation, FL, confidentiality, active defense	Learning/security overhead is rarely co-evaluated with orchestration and agricultural outcomes	Indirect enabling evidence
Agricultural connectivity, edge, and offloading	[36,37,38,39,40,41,42,43,44,45,46]	Rural access, UAV/satellite links, edge/cloud processing, task offloading	Controller placement and NFV/SFC lifecycle management are not the primary focus	Agriculture direct; SDN/NFV limited
Phenotyping, digital twins, and smart breeding	[47,48,49,50,51]	Phenotypic data, twin synchronization, growth/breeding decisions	Weak mapping to dynamic cross-domain communication/computing orchestration	Agriculture direct; SDN/NFV limited
Position of this review	-	SDN/NFV-enabled cross-domain resource management mapped to agricultural workloads and outcomes	Common comparison of objectives, decisions, gains, costs, and evidence boundaries	Joint focus

**Table 2:** Characteristics, resource constraints, and resource-management pressures across the three SAGIN layers.

Network Layer	Main Technical/Resource Characteristics	Typical Resource Constraints	Direct Resource-Management Pressure
Space segment (e.g., LEO)	Wide-area coverage, orbital motion, inter-satellite/satellite-ground links, limited onboard computing and storage	Visibility windows, propagation/link latency, payload capacity, continuously changing topology	Dynamic association and handover, controller placement, inter-satellite resource scheduling, service continuity
Aerial segment (UAV/HAP)	On-demand deployment, controllable positioning, relay/edge-computing capability	Flight energy, payload/computing capacity, backhaul links, residence time	Trajectory-communication-computing cooperation, task offloading, temporary coverage, elastic services
Terrestrial segment	High-capacity access, stronger computing/storage, fixed/mobile edge facilities	Local coverage, hotspot congestion, disaster failures, heterogeneous cross-domain interfaces	Traffic scheduling, edge-resource orchestration, cross-domain state aggregation, service assurance

**Table 3:** Input–processing–output characteristics and applicability of SDN controller-placement algorithms.

Category/Representative References	Input -> Processing -> Output	Applicability, Strengths, and Limitations
Heuristic iteration [19]	LEO topology, latency, capacity, availability -> initial placement + Z-score local exchange -> controller count/locations and node association	Transparent and easy to implement; useful as an approximate baseline, but local search does not guarantee global optimality, and reliability is score-based.
Clustering/partitioning [17,18]	Position/orbit/traffic/distance -> K-Means++ or improved K-Means partitioning -> control domains, representative controllers, dynamic associations	Compresses the search space and improves scalability; suitable for periodic reconfiguration, but controller count must be determined, and repeated clustering incurs overhead.
Metaheuristic/hybrid [16,17,74,75]	Topology, load, capacity, multi-objective state -> SA/Tabu/GA/ISAO search -> near-global placement, association, migration/switching plan	Handles non-convex multi-objective CPPs and escapes local optima, but is parameter sensitive and incurs higher iterative and migration cost.
Exact/stochastic optimization [72,73]	Candidate locations, request rates, service rates, link/probability parameters -> ILP/CCSP/two-stage stochastic programming -> controller number, locations, static/adaptive associations	Auditable objectives and constraints; valuable as a benchmark, but costly at scale. The two core sources are wireless/cellular SDN rather than SAGIN-specific models.

**Table 4:** Comparison of SFC deployment and orchestration solution mechanisms in SAGIN.

Category/Representative References	Core Mechanism	Main Objective	Applicability Boundary/Limitation
Exact mathematical-programming baselines [20,21,80,81,82]	ILP/MILP constraint modeling; can be solved with tools such as Gurobi	Latency, profit, resource balance, revenue/resource cost, recovery time	Useful for feasible-region and optimality baselines; larger problems usually require approximation, decomposition, or learning.
Heuristic/search [21,80,81]	Tabu Search, multidimensional weighted paths, low-complexity multicast embedding	Online profit, resource balance, deployment cost	Depends on neighborhoods, weights, and candidate paths; quasi-static assumptions and search budgets affect online applicability.
Matching/game [24,82]	Three-sided stable matching; failure-recovery matching	Demand compatibility, acceptance rate, recovery time, service continuity	Sensitive to preference functions and matching scale; overhead of large dynamic matching must be evaluated.
Learning-driven [22,23,83,84]	MDP/DRL, D3QN/adversarial learning, spatiotemporal actor–critic, federated embedding	Number of successful deployments, long-term profit, lifecycle benefit	Training, inference, and synchronization overhead; sensitive to state, reward, and topology-distribution shifts.

**Table 5:** Comparison of representative cooperative SDN/NFV resource-management mechanisms.

Cooperation Mechanism/Representative References	Control-Virtualization Coupling	Main Benefit/Objective	Applicability Boundary and Cost
State sharing and QoS-aware VNE [25]	SDN aggregates cross-domain CPU, bandwidth, and latency states; RL performs node mapping; k-shortest path performs link mapping	Adapts resource preference by QoS class while balancing resource efficiency, acceptance, and low-latency requirements	Node prefiltering may reduce utilization; cross-domain state reporting adds signaling and computation.
Dynamic mapping and rescheduling [21]	Real-time state triggers VNF migration, re-instantiation, and execution-order adjustment; MILP + Tabu Search for online approximation	Improves cumulative profit while satisfying service latency/order constraints	Migration cost, additional latency, and online-search overhead interact.
Multi-party demand–resource matching [24]	Scenario and multi-attribute resource states support three-sided stable matching among devices, user groups, and vehicular users	Improves demand compatibility, acceptance, and cost efficiency	Sensitive to preference construction and dynamic matching scale; cross-domain state-representation cost must be evaluated.
Closed-loop QoE/QoS orchestration [26]	Centralized GEO control + on-demand LEO VNF instantiation; MDP closes state, QoE/QoS, and OPEX	Improves end-to-end QoE under QoS constraints while controlling operational expenditure	LSTM history increases inference latency; fixed QoE weights and state synchronization limit generalization.

**Table 6:** Representative learning-driven SAGIN mechanisms and their main deployment limits.

Category/Representative Reference	Learning and Decision Structure	Representative Objective/Result	Main Deployment Boundary
Hierarchical FL and topology optimization [27]	Terrestrial training + LEO aggregation + GEO routing; TAEER solves DST	Reduce aggregation energy while maintaining routing reliability	DST is NP-hard; topology/link-state sensitivity and energy-reliability trade-off
HFL + hybrid-action DRL [28]	UAV edge + LEO cloud aggregation; H-DSAC handles mixed actions	Balance multi-task performance and fairness within limited service time	Complex training, replay/update overhead, and multi-level synchronization
Single-agent joint-scheduling DRL [29]	Queue state -> offloading, processing, bandwidth, and CPU actions	Joint latency/energy optimization with dropped-task penalty	State/action growth with tasks and nodes; long training stabilization
Distributed DRL [31]	Local server per domain; offline training and online mapping with partial sharing	Improve utilization, acceptance, and revenue/cost performance	Baseline sensitive; distributed control still incurs state-sharing overhead
Cooperative MARL [30]	Per-UAV actors + centralized critic (CTDE); joint offloading/bandwidth decisions	Maximize reliable offloading under QoS constraints	Training cost grows with agent count; trajectory decisions remain outside the model

**Table 7:** Representative SAGIN security mechanisms and their principal evidence boundaries/resource costs.

Mechanism/Representative Reference	Security Decision, Evidence, and Main Boundary
Physical-layer confidentiality [33]	Two-hop eavesdropping; jointly optimize satellite power and UAV beamforming for secrecy rate. CObI/HbSAI outperform the baseline, but CObI is computationally intensive and HbSAI remains heuristic; conclusions depend on the assumed channel/two-hop model.
SFC active defense [34]	HoneyVNF deception + PPO deployment + moving-target/local migration improves SFC security while limiting acceptance/resource penalties. HoneyVNFs and migration still consume CPU, storage, bandwidth, and reconfiguration time.
Trusted spectrum transactions [35]	Digital-twin state mapping + knowledge-graph recommendation + smart-contract execution improves transaction/spectrum-use outcomes. Validation does not establish resilience to major blockchain attacks, and sparse knowledge graphs can degrade recommendations.
GAI security enhancement [14]	GAN/VAE/GDM/LLM techniques support synthesis, reconstruction, and policy generation across security objectives. Evidence is survey-level; compute/data demand, poisoning, inversion, adversarial prompts, hallucination, and distribution shift remain major costs/risks.
General intelligent IDS support [32]	IoT IDS taxonomies provide mechanism-level guidance for centralized/distributed detection, but their accuracy cannot be transferred directly to SAGIN; distributed IDS also increases node energy, storage, and computation.

## Data Availability

No new data were created or analyzed in this study. Data sharing is not applicable to this article.
